# Physical Conditions of Fast Glacier Flow: 3. Seasonally‐Evolving Ice Deformation on Store Glacier, West Greenland

**DOI:** 10.1029/2018JF004821

**Published:** 2019-01-30

**Authors:** T. J. Young, P. Christoffersen, S. H. Doyle, K. W. Nicholls, C. L. Stewart, B. Hubbard, A. Hubbard, L. B. Lok, P. V. Brennan, D. I. Benn, A. Luckman, M. Bougamont

**Affiliations:** ^1^ Scott Polar Research Institute University of Cambridge Cambridge UK; ^2^ British Antarctic Survey, National Environmental Research Council Cambridge UK; ^3^ Centre for Glaciology, Department of Geography & Earth Sciences Aberystwyth University Aberystwyth UK; ^4^ Centre for Arctic Gas Hydrate, Environment and Climate, Department of Geology Arctic University of Norway Norway; ^5^ Department of Engineering Lancaster University Lancaster UK; ^6^ Department of Electronic & Electrical Engineering University College London London UK; ^7^ School of Geography & Sustainable Development University of St. Andrews St. Andrews UK; ^8^ Department of Geography Swansea University Swansea UK

**Keywords:** Greenland, Glacier, Radar, Strain, Ice Sheet

## Abstract

Temporal variations in ice sheet flow directly impact the internal structure within ice sheets through englacial deformation. Large‐scale changes in the vertical stratigraphy within ice sheets have been previously conducted on centennial to millennial timescales; however, intra‐annual changes in the morphology of internal layers have yet to be explored. Over a period of 2 years, we use autonomous phase‐sensitive radio‐echo sounding to track the daily displacement of internal layers on Store Glacier, West Greenland, to millimeter accuracy. At a site located ∼30 km from the calving terminus, where the ice is ∼600 m thick and flows at ∼700 m/a, we measure distinct seasonal variations in vertical velocities and vertical strain rates over a 2‐year period. Prior to the melt season (March–June), we observe increasingly nonlinear englacial deformation with negative vertical strain rates (i.e., strain thinning) in the upper half of the ice column of approximately −0.03 a^−1^, whereas the ice below thickens under vertical strain reaching up to +0.16 a^−1^. Early in the melt season (June–July), vertical thinning gradually ceases as the glacier increasingly thickens. During late summer to midwinter (August–February), vertical thickening occurs linearly throughout the entire ice column, with strain rates averaging 0.016 a^−1^. We show that these complex variations are unrelated to topographic setting and localized basal slip and hypothesize that this seasonality is driven by far‐field perturbations in the glacier's force balance, in this case generated by variations in basal hydrology near the glacier's terminus and propagated tens of kilometers upstream through transient basal lubrication longitudinal coupling.

## Key Points

- Autonomous phase‐sensitive radar can be used to track the fine‐scale motion of glacier internal layers
- Vertical velocity profiles of internal layers evolve seasonally in a region of fast flow
- Changes in the local strain field are driven by downstream far‐field longitudinal stress coupling

## 1 Introduction

Conditions at the ice‐bed interface exert a major control on the force balance of glaciers and ice sheets. Spatially, the flow of ice masses is modulated by variations in basal traction, which arise from variations in bed topography (Gudmundsson, 1997, 2003), hydrology (Hoffman et al., 2016), the presence and properties of sediments (Bougamont et al., 2014), and the basal thermal regime (Christoffersen & Tulaczyk, 2003; Tulaczyk et al., 2000). In places where soft subglacial sediments provide limited frictional resistance to ice flow, the gravitational driving stress may be countered predominantly by lateral stresses along the glacier's side walls (Raymond, 1996; Rippin et al., 2005), or by membrane‐like stresses (Hindmarsh, 2006), consisting of longitudinal and transverse stress gradients within the ice mass itself (Kamb & Echelmeyer, 1986; Price et al., 2008).

Variations in basal traction can also be temporal, with fluctuations in subglacial water pressures causing ice flow to vary on timescales from hours to months (e.g., Bartholomaus et al., 2008; Doyle et al., 2015; Hooke et al., 1989; Iken & Bindschadler, 1986; Iken & Truffer, 1997; Kamb, 1987; Meierbachtol et al., 2016). Such changes in local and regional water pressures can occur when there is a sudden influx of water into the basal environment, either from the surface where meltwater is produced with diurnal variations (Bartholomew et al., 2012) or released from surface lake drainage (Doyle et al., 2014; Hoffman et al., 2011) or at the bed from an active subglacial lake (Fricker & Scambos, 2009; Palmer et al., 2015; Wingham et al., 2006).

Although the concept of force balance is well established in the geophysical and glaciological literature, it is rarely measured and mostly applied within numerical modeling studies (Kamb & Echelmeyer, 1986; Meierbachtol et al., 2016; Price et al., 2008). Direct measurements of depth‐dependent englacial strain are important because they inform how ice deforms internally when glaciers flow. These measurements are, however, sparse as they typically require installation of strain gauges and inclinometers in boreholes, which are both costly and time‐consuming (Gudmundsson, 2002; Keller & Blatter, 2012; Paterson, 1976; Perutz, 1952; Raymond et al., 1994; Ryser, Lüthi, Andrews, Hoffman, et al.,^2014^; Sugiyama & Gudmundsson, 2003).

Phase‐sensitive radio‐echo sounders (pRES) provide the first noninvasive, direct, and continuous measurements of vertical velocity and englacial strain (Brennan et al., 2014). Pioneered by Corr et al. (2002), the instrument has primarily been used to measure basal melt rates of Antarctic ice shelves with its ability to detect range with millimeter precision by repeat stake surveys (Jenkins et al., 2006, 2010; Marsh et al., 2016; Stewart, 2018). Jenkins et al. (2006) examined and discussed spatial and temporal variations in vertical strain rates in detail, observing force balance effects of tidal bending, while within other studies, measurements of vertical strain rates were an intermediary result to obtain basal melt rates beneath Antarctic ice shelves. Of the few studies conducted on grounded ice sheets, focus on the vertical movement of internal layers was limited to ice divides, measuring strain rates on the order of 1× 10^−4^a^−1^ as a result of slow flow. Gillet‐Chaulet et al. (2011) tracked englacial layers along two 20‐km survey lines crossing ridges near the GRIP and NEEM ice‐core sites to quantify the rheological properties of ice near divides. Kingslake et al. (2014) and Kingslake et al. (2016) used a similar survey design to investigate the Raymond (1983) Effect near ice divides. In 2014, the development of a low‐power autonomous pRES (ApRES) allowed long‐term, unattended monitoring of ice‐shelf and ice‐sheet thinning (Nicholls et al., 2015). Importantly, this new instrument allowed measurement of vertical strain rates on fast‐flowing glaciers and ice streams due to its ability to track unambiguously and continuously the vertical movement of deforming internal layers at sufficiently short time steps (Young et al., 2018).

Two companion studies conducted simultaneously on Store Glacier have investigated and characterized in detail the ice‐bed interface and the subglacial environment, providing insight into the conditions and mechanisms of fast glacier flow. Through boreholes drilled and instrumented with englacial and subglacial sensors during the summers of 2014 and 2016, supplemented with surface velocity and meteorological measurements, Doyle et al. (2018) observed a steeply curving vertical temperature profile characteristic of fast flow, with basal motion accounting for 60–70% of the measured surface horizontal velocities and internal deformation, largely within the lowermost ∼100 m of the ice column accounting for the remaining 30–40% of the mean annual flow rate of ∼600 m/a. The fast basal motion is thought to be facilitated by a spatially extensive and persistently inefficient subglacial drainage system. Concurrently, orthogonal seismic profiles conducted by Hofstede et al. (2018) at the same study site revealed extensive (∼45‐m‐thick) unconsolidated sediments underlying the 600‐m‐thick ice, accompanied by patches of variable basal slipperiness associated with the presence of water, suggesting low, but spatially variable ice‐bed coupling.

This study extends the scope of Doyle et al. (2018) and Hofstede et al. (2018) by investigating the internal deformation of the ice column as a result of fast ice flow. Spatially and temporally coincident with the two previous studies, we used ApRES to measure daily displacements of internal reflectors within Store Glacier, West Greenland. The records, which have millimeter accuracy and cover the majority of a 2‐year period, are the first to resolve the fine‐scale change in englacial strain in a fast‐flowing outlet glacier of the Greenland Ice Sheet. We observe the seasonal evolution of the vertical strain profile and investigate the causes of these variations using complementary borehole data acquired in the close vicinity of the ApRES deployments.

## 2 Study Area

**Figure 1:**
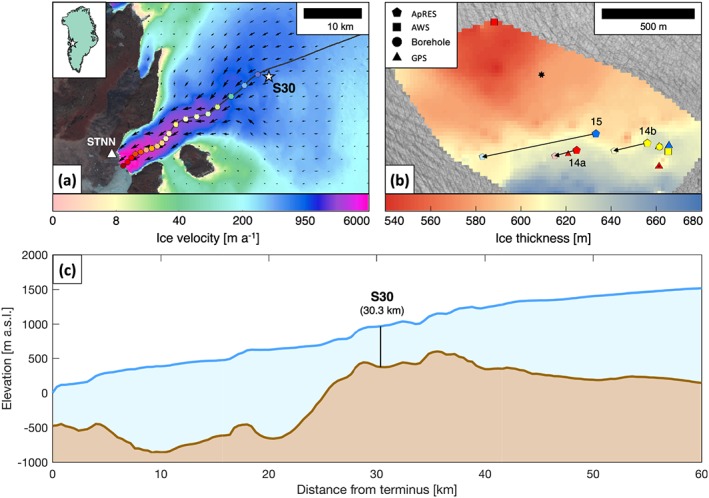
(a) The 2015–2016 averaged InSAR‐derived surface velocities of Store Glacier (Joughin et al., 2010), with the location of S30 and corresponding sampled points from TerraSar‐X velocity measurements in Figure 8a along the central flowline (spaced 1–3 km apart). STNN shows the location of the basestation GPS used to reference kinematic GPS data. (b) Overview of S30 showing location and trajectory of deployments with arrows showing displacements of ApRES units over their respective operational periods (Figure 5) underlain by an interpolated ice thickness map measured using separate ApRES surveys. Colors represent starting time of deployment (red = May 2014; yellow = August 2014; blue = July 2015). The black seven‐pointed star references the approximate location of a basal “sticky spot” as observed by Hofstede et al. (2018). (c) Surface and bed topography of Store Glacier along the central flowline calculated from Greenland Ice Mapping Project digital elevation model (Howat et al., 2014) and BedMachine v3 (Morlighem et al., 2017), respectively. ApRES = autonomous phase‐sensitive radio‐echo sounder.

Store Glacier (Qarassap Sermia) is the third fastest outlet glacier in West Greenland (Figure 1a), draining a catchment of 35,000 km^2^. At the terminus, the glacier is 5 km wide, flows at 6,300 m/a (Rignot & Mouginot, 2012), and discharges 14–18 km^3^ of ice into the ocean annually (Weidick, 1995). While many surrounding glaciers have recently experienced dynamic thinning due to acceleration and retreat of their termini, Store Glacier has been observed to be stable in both mass budget and terminus position since at least 1968 with a 200‐m seasonal oscillation (Box & Decker, 2011; Howat et al., 2010; Weidick, 1995). This stability is underpinned by topographic narrowing and grounding on a sill that is ∼450 m below sea level near the glacier's terminus; however, past this sill, the bed is retrograde 30‐km inland from the margin, with depths reaching as deep as 900 km below sea level (Figures 1a and 1c; Morlighem et al., 2016).

Our study site, S30, is located ∼30‐km inland of Store Glacier's calving front and within ∼1 km of its central flowline where the ice is 600–650 m thick (Figure 1b). Here the glacier exhibits moderate changes in summer ice velocities, increasing from ∼600 m/a in winter to ∼700 m/a in summer, when surface meltwater is transferred to the subglacial environment (Doyle et al., 2018).

## 3 Methods

### 3.1 ApRES and Antenna Array Installation and Architecture

During three field campaigns (May 2014, July/August 2014, and July 2015), we deployed three ApRES arrays (labeled 14a, 14b, and 15, respectively) at site S30 on Store Glacier (Figure 1a). At each deployment site, the arrays were anchored to the ice to maintain continuous contact with the ablating surface. The ApRES arrays flowed downglacier at a rate of ∼600 m/a, each continuously recording the depth of internal reflectors relative to the array and tracking their vertical movement through the incident ice column within a Lagrangian reference frame (i.e., measurements spatially referenced to the array). As the ice surface ablated during the summer melt season, the frame of the ApRES array (supporting information Figure S1) maintained continuous contact with the surface by lowering under its own weight down poles drilled 3 m into the ice surface. In this way, drifting snow occurring outside the winter months would only accumulate around and not underneath the radar and therefore would not impact range detection of internal layers due to the array being anchored to the ice surface in summer. Any surface ablation, and therefore lowering of the radar array, would register as a constant offset where internal layers collectively shift toward the antennas (Figure S2). Because the calculation of vertical displacement and deformation is relative between internal layers, the effects from surface ablation have no impact on the data processing procedures.

The three array sites were separated from each other by up to 450 m and made measurements at a sampling interval ranging from 1 to 8 hr (Figure 1a and Table 1). Deployment 15 (blue pentagon, Figure 1b) operated continuously for 338 days from 05 July 2015 to 06 June 2016 and traveled 570 m downglacier, while deployments 14a and 14b (red and yellow pentagons, respectively, Figure 1b) operated for 72 and 124 days and traveled 121 and 213 m, respectively. Deployment 14b ended abruptly on 04 December 2014 when the array was damaged by strong winds. Deployments 14a and 15 were manually terminated, the former being moved upglacier to align with the drill site (Doyle et al., 2018), and the latter disassembled in concordance with the conclusion of the project. Data collected from each deployment were collapsed into one dimension. Further information of the array design used in the field deployments are detailed in supporting information S1 and in Young et al. (2018).

**Table 1:** Metadata on the Three Autonomous Phase‐Sensitive Radio‐Echo Sounders Instruments and Settings Deployed at S30

Deployment	14a	14b	15
Start location (latitude)	70° 31^*′*^ 2^*′′*^ N	70° 31^*′*^ 5^*′′*^ N	70° 31^*′*^ 7^*′′*^ N
Start location (longitude)	49° 55^*′*^ 46^*′′*^ W	49° 55^*′*^ 9^*′′*^ W	49° 55^*′*^ 37^*′′*^ W
Start Altitude (m a.s.l.)	961	981	961
Start date/time (UTC)	2014‐05‐06 20:46	2014‐08‐03 17:55	2015‐07‐05 19:40
Stop date/time (UTC)	2014‐07‐16 12:42	2014‐12‐04 02:05[Link]	2016‐06‐06 08:25^a^
Duration (days)	72	124	338
Distance traveled (m)	121	213	570
Burst mode	MIMO	MIMO	Alternating^b^
Burst interval (hr)	1	4	8^b^
Total bursts	1,668	734	1,011^c^
Chirps per burst	64	64	64
Chirp sampling rate (Hz)	40,000	40,000	40,001
Tx_1_‐Rx_1_ separation (mm)	2,350	2,700	2,480
Orientation^d^ (°)	+12	0	0
Data processing depth range (m)	20–590	20–590	20–620

*Note*. a.s.l = above sea level; MIMO = multiple‐input multiple‐output.

a Termination due to instrument hardware failure.

b Alternate bursts between quasi‐monostatic and MIMO mode every 4 hr.

c Per burst mode.

d Relative to the principal flow direction (262°).

From these records, we measured and modeled the vertical displacement and velocity of these reflectors relative to the starting location of the ApRES antennas and calculated vertical strain‐rate profiles from time series of the vertical velocity of internal reflectors. Through this process, we standardized the time step of each time series by downscaling the temporal resolution to 24 hr, resulting in continuous daily measurements of ice column vertical deformation.

**Figure 2:**
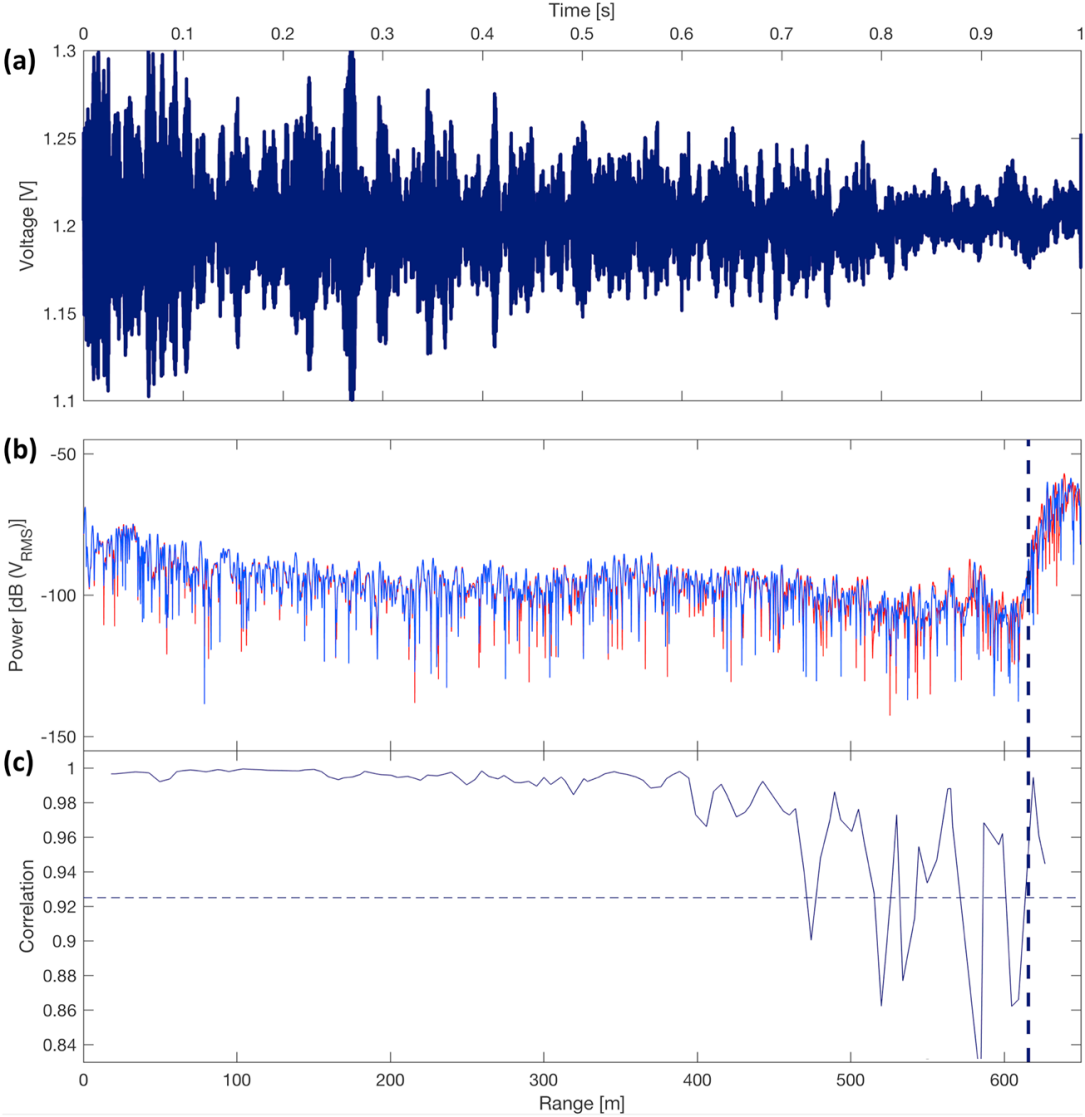
Phase processing of two paired autonomous phase‐sensitive radio‐echo sounders burst‐mean samples (profiles obtained 19:34 4 November 2014 UTC and 19:34 5 November 2014 UTC). (a) Raw time series of averaged burst consisting of 64 chirps. (b) Fourier transform of first (red) and second (blue) averaged bursts. (c) Coherence of cross correlation of segments, which represent internal reflectors. Only reflectors above the coherence threshold of 0.925 (thin dotted line; Figure S4b) were used in subsequent analyses. The thick dotted line identifies the ice‐bed interface.

Each ApRES unit uses the frequency‐modulated continuous‐wave (FMCW) technique, where a carrier wave is continuously transmitted over a spectrum of frequencies, sampling over periods much greater than the target two‐way travel time (Nicholls et al., 2015). The carrier signal linearly sweeps between 200 and 400 MHz with a center frequency of 300 MHz (corresponding to a coarse range wavelength resolution of *λ*_*c*_ = 0.43 m in cold ice), forming a “chirp,” with an ensemble of chirps known as a “burst” (Figure 2a). This technique allows for sufficient ice penetration, while its phase sensitivity allows it to achieve millimeter‐depth precision (e.g., 3‐mm root mean square precision at 1.8‐km range; Brennan et al., 2014). Crucially, the power consumption of the system is on the order of 5 W during operation and 1 mW while in standby, which, with an on/standby duty cycle of ∼1 min every 6 hr, gives a mean power consumption of 31 mW (Brennan et al., 2014). With each radar deployment configured to sample 64 chirps per burst, a continuous year‐long autonomous operation with a frequency of six bursts per day can be achieved using one 6 V, 180‐Ah battery, even after appropriate derating of the battery to allow for low temperatures.

### 3.2 ApRES Measurements of Vertical Deformation

**Figure 3:**
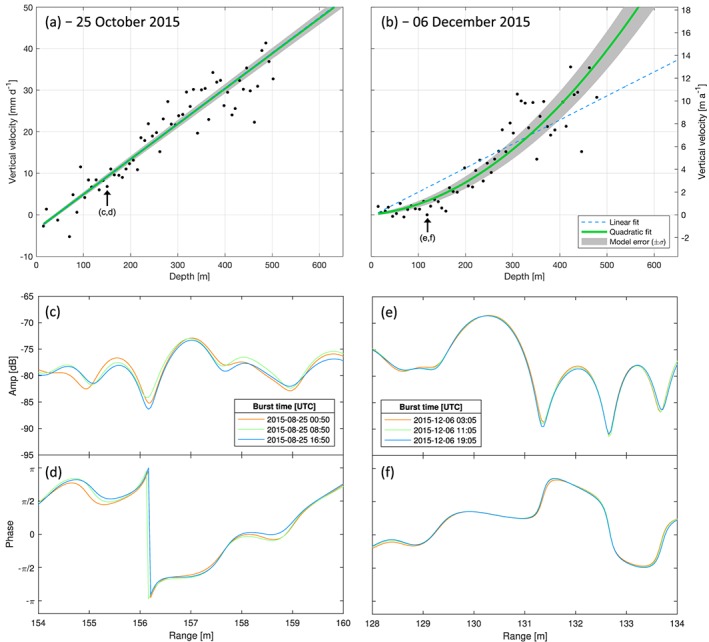
(a and b) Examples of two autonomous phase‐sensitive radio‐echo sounder‐measured vertical velocity profiles fitted with a weighted robust quadratic regression (green; equation (10)), with the model error a function of curve fitting. A weighted robust linear regression (dashed blue) is overlain as comparison. Each marker represents one englacial reflector; all reflectors are relative to the initial surface of the measured ice column. (c) and (d), respectively, show the amplitude and phase of the specified internal reflector identified in (a); (e) and (f) show equivalent plots for the specified internal reflector identified in (b).

**Figure 4:**
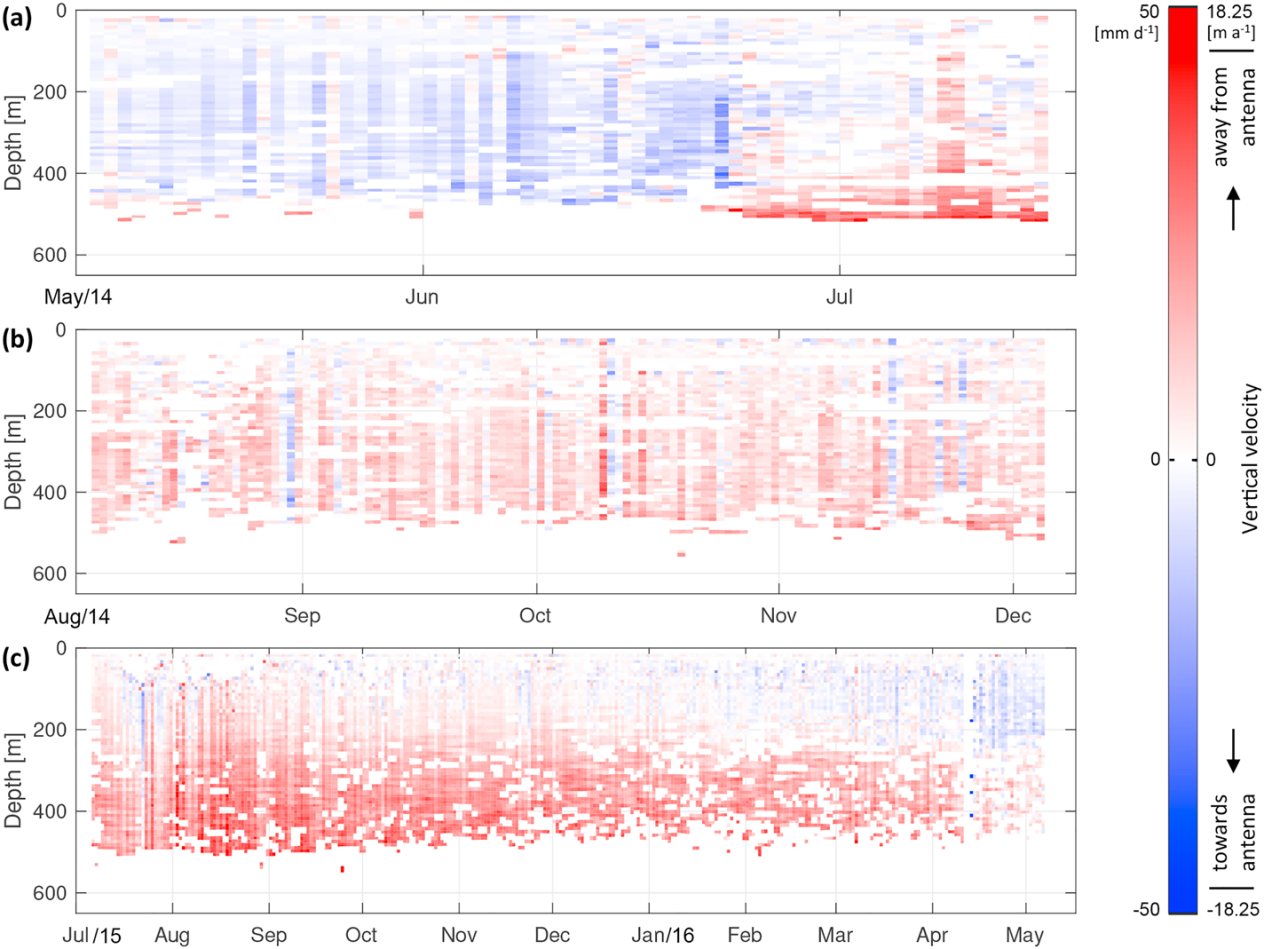
Vertical velocity time series relative to the transmitting/receiving antenna for internal reflectors within the entire ice column measured using autonomous phase‐sensitive radio‐echo sounders at S30 from deployments (a) 14a; (b) 14b; and (c) 15. Here blue represents strain thinning and red strain thickening. The effects of surface ablation on internal reflector vertical velocities were removed (see supporting information S1).

The ApRES, when left in unattended mode, measures the vertical motion (the vertical velocity) of individual internal reflectors relative to the radar antennas by comparing their internal phase between successive bursts (Figure 3; Nicholls et al., 2015). Then, to examine how vertical strain varies through time, we applied best fits with quadratic models (Figure 3) to each daily velocity profile (i.e., Figure 4 to S6) and calculated vertical strain rates (i.e., Figure S6 to 5) through taking the derivative along each modeled profile).

**Figure 5:**
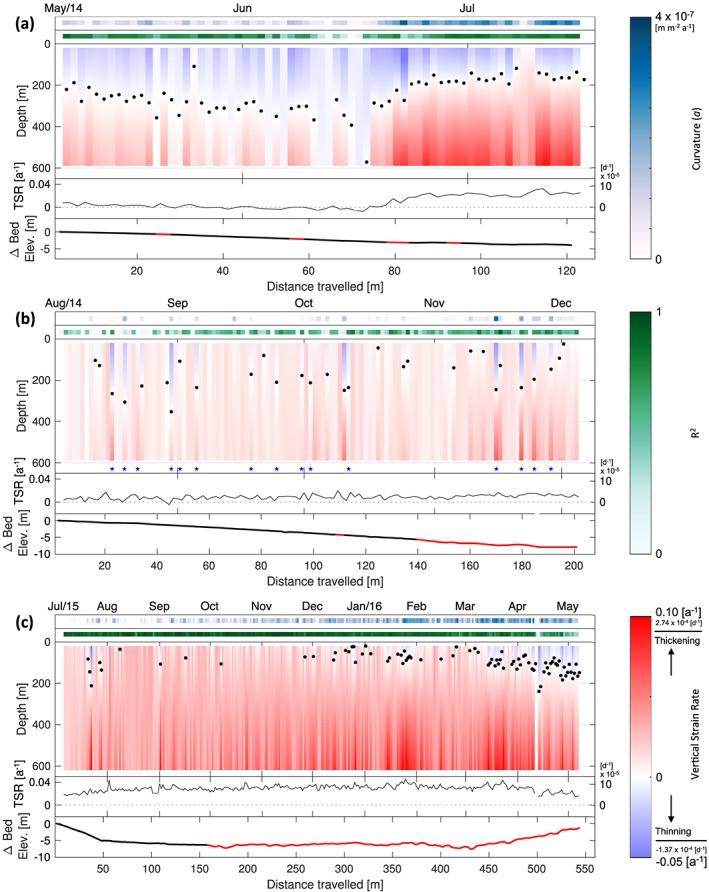
Time series of modeled internal layer vertical strain rate profiles within the ice column measured using autonomous phase‐sensitive radio‐echo sounders at S30 at sites (a) 14a; (b) 14b; and (c) 15. The horizontal bars at the top of each plot show the curvature of the vertical velocity profile (parameter a, white–blue color scale) and the goodness‐of‐fit (R ^2^, white–green color scale) of the output model for each day of observation. The location of black dots signifies the depth at which the ice column switches in polarity from a vertically compressive (i.e., vertical strain thinning, in blue) to a vertically extensive (i.e., vertical strain thickening, in red) regime. Within (b), blue stars mark ephemeral bursts of anomalous strain (R ^2^ > 0.5), coincident with the time series of basal traction (Figure 7d). A contemporaneous time series of the TSR is shown below each deployment strain rate record. The bed elevation for each transect (black) was calculated by subtracting the local ice thickness (measured by autonomous phase‐sensitive radio‐echo sounders) from GPS measurements of surface elevation. A surface digital elevation model (Greenland Ice Mapping Project digital elevation model; Howat et al., 2014) was substituted where there are gaps in GPS data (red). Note the different scales used for time and distance between subplots. TSR = total strain rate.

#### 3.2.1 Determination of Vertical Velocity Profiles

To calculate the vertical displacement of internal reflectors and their associated phase noise, we follow the (i) phase processing procedures described by Brennan et al. (2014; Figure S3a) and the (ii) phase differencing procedures described by Stewart, (2018; Figure S3b), thereby producing time series of one‐dimensional depth profiles of vertical velocity spanning the majority of a 2‐year period (2014–2016).

The phase processing procedure consists of (i) weighting the deramped waveform in the time domain using a Blackman window; (ii) increasing the series to a multiple of its original length by appending trailing zeros (“zero padding”); (iii) time shifting the signal so that the phase center aligns with the start of the sample; and (iv) Fast Fourier‐Transforming (FFT) the modified time series to transform the waveform into the frequency domain. Due to the proportionality of frequency and time‐of‐flight in deramped frequency‐modulated continuous‐wave observations (equation 1 of Brennan et al., 2014), the resulting complex‐valued spectrum is now a range‐domain representation of the returned signal (Figure 2b) analogous to a time‐domain radar profile (Figure 2a). The system generates a deramped frequency of 2.35 Hz/m, equivalent to a coarse range bin (the wavelength) of 0.43 m assuming a constant propagation speed of 170 × 10^6^ s^−1^ (Brennan et al., 2014). The amplitude of each Fourier coefficient represents the net strength of reflectors within the range bin. From this, the temporal difference between the transmitted and received signals gives the “coarse range” (*R*_*c*_), whereas the phase provides an indication of the “fine range” (*R*_*f*_) of the reflector within the specified coarse range bin *n* through the following relations (equations 1 and 15 of Brennan et al., 2014):

(1) $$R_{c}\left(n\right)=\frac{nc}{2B\sqrt{\varepsilon_{r}}p},$$

(2) $$R_{f}\left(\text{mod}\;\frac{\lambda_{c}}{2}\right)=\frac{{\lambda {}_{c}\phi }_{c}}{4\pi },$$

where multiple reflectors are present within the range bin that influence the observed amplitude and phase through their coherent sum. Here the fine range *R*_*f*_ gives a precise measurement relative to the specified coarse range bin center *n* if constrained to $\left[\pm \lambda_{c}/4\right]$, where *λ*_*c*_ is the center wavelength of the ApRES system (1 m). In equation (1), *B* is the system bandwidth (200–400 MHz = 200 MHz), *c* is the speed of light, *p* is the pad factor, and *ε*_*r*_ is the dielectric constant of ice, which we assume to be 3.1 (Brennan et al., 2014). The pad factor determines the amount of zero padding to be applied by lengthening the time sample by a multiple of *p*, thereby reducing the spacing between range bins by the same factor (Δ*R*_*c*_/*p*). All data processed here used a pad factor of 10; variation of the pad factor did not significantly alter results. In equation (2), ϕ_*c*_ is the phase of the deramped signal at the coarse range bin center. The sum of the coarse range and fine range will, when the above conditions are satisfied, give an estimate of the total range from the radar to the specified reflector, which is precise to ∼3‐mm root mean square at a distance of 1.8 km (Brennan et al., 2014):

(3) $$R\left(n\right)=R_{c}\left(n\right)+R_{f}\left(n\right).$$

In equation (2), a range‐dependent phase offset was applied to the signal so that the phase observations (already FFT‐processed and deramped) are zeroed at the bin center (ϕ_*c*_) rather than at the antenna (ϕ_*d*_). This phase shift is essential if offsets are calculated using phase differencing procedures to determine changes in range to millimeter precision. Mathematically, the amount of phase shift is represented by weighting the FFT‐processed deramped waveform by a reference array equal to the phase conjugate of the expected phase at the center of the range bin. The reference array is described by equation 17 of Brennan et al. (2014):

(4) $$\phi_{\text{ref}}\left(n\right)=\exp\left[-i\left(\frac{n\omega_{c}}{Bp}-\frac{n^{2}K}{2B^{2}p^{2}}\right)\right].$$

Here *ω*_*c*_ is the chirp center frequency in terms of angular velocity (rad/s), *K* = 2*πB*/*T* is the chirp gradient (rad/s^2^) that represents the frequency‐modulated sweep rate, where, within the equation, *B* is the system bandwidth (200–400 MHz), and *T* is the total pulse duration.

For the phase differencing procedure, burst pairs from each range‐processed spectrum (profile) were then processed iteratively with a daily (24‐hr) time step through complex cross correlation of the burst spectra pair (Rosen et al., 2000; Stewart, 2018). Phase differences were calculated by comparing bursts sampled 24 hr apart using short (5‐m) segments along the profile, where each segment represents the internal reflectors residing within the segment depth range. With sampling intervals averaging from 1 to 8 hr, this provided 3–24 estimates of reflector displacement per day, and daily average velocities were calculated from the output.

The amount of vertical displacement for each segment (Figure 2b) was calculated by processing the complex cross correlation of the segment in the first profile *f* with the corresponding segment in the second profile *g* (equation 4.18 of Stewart, 2018):

(5) $$f⋆g\left(m,n,\Lambda \right)=\frac{\sum f^{*}\left(k\left(m,n\right)\right)g\left(k\left(m,n\right)+\Lambda \right)}{\sqrt{\sum {\left|f\left(k\left(m,n\right)\right)\right|}^{2}}\sqrt{\sum {\left|g\left(k\left(m,n\right)+\Lambda \right)\right|}^{2}}}.$$

Here the complex correlation *f*⋆*g* is the multiplication of the profile *g*, lagged with the vector Λ, with the complex conjugate of profile *f*, indicated by ^∗^. $k\left(m,n\right)=\left[k_{-n}\text{⋯}k_{-2}k_{-1}k_{0}k_{1}k_{2}\text{⋯}k_{n}\right]$ represents an array of range bin indices that surround the center of the specified range segment *m* = *k*_0_. While the length of ***k*** has no impact on the performance of the complex correlation, ***k*** should be sufficiently long (i.e., containing enough sample reflectors for accurate cross correlation) to correctly identify the amount of displacement between reflector pairs. The magnitude of *f*⋆*g* is the coherence, which indicates the similarity of the segments to each other, and is scaled from 0 for incoherent segments to 1 for segment pairs with a constant phase difference. The phase of *f*⋆*g* is the amplitude‐weighted (vector‐averaged) phase difference between the segments.

In cross correlation, the goal is to maximize the coherence, which then identifies the most‐likely amount of displacement: ${\hat{\gamma }}_{fg}\left(n,\hat{\Lambda }\right)=\underset{\Lambda }{\arg\max}\;\gamma_{fg}\left(n,\Lambda \right)$. Here the maximization of the coherence and its corresponding lag vector is represented by the hat operator ^. Therefore, the coarse‐range displacement of the segment *n* is the amount of lag $\hat{\Lambda }$ that corresponds to the maximum coherence ${\hat{\gamma }}_{fg}$, scaled by the coarse‐range resolution (equation (1)):

(6) $$\delta_{c}\left(n\right)=\Delta R_{c}\Lambda \left(n\right),$$

where the maximum coherence of *f*⋆*g* is

(7) $${\hat{\gamma }}_{fg}\left(n,\hat{\Lambda }\right)=\underset{\Lambda }{\arg\max}\;\gamma_{fg}\left(n,\Lambda \right)=\underset{\Lambda }{\arg\max}\left|f⋆g\left(m,n,\Lambda \right)\right|.$$

Similarly, the fine‐range displacement of the segments is calculated by substituting φ_*c*_ for $\hat{\Lambda }$ in the equation calculating the referenced and centered phase within the coarse range bin (equation (2)):

(8) $$\delta_{f}\left(n\right)=\frac{\lambda_{c}\hat{\Lambda }}{4\pi }.$$

Finally, the total displacement of the segments is the sum of the coarse‐ and fine‐range displacements:

(9) $$\delta_{t}\left(n\right)=\delta_{c}\left(n\right)+\delta_{f}\left(n\right).$$

#### 3.2.2 Modeling Full‐Column Vertical Strain

Depth‐dependent profiles of vertical strain were calculated by fitting a model to each internal layer's vertical velocity profile. As these profiles vary seasonally and on timescales as short as a single day, we used quadratic best‐fit models (Figures 3a and 3b) to describe englacial strain rates in the form:

(10) $$\delta_{m}\left(R\right)=aR^{2}+bR+c.$$

Here $\delta_{m}\left(R\right)$ is the modeled displacement, and the model parameters *a*, *b*, and *c* were solved using a robust nonlinear least‐squares search. The use of a quadratic model, though simple, easily identifies the degree of nonlinearity within vertical velocity profiles through the first model parameter *a*, which represents the curvature of the model with depth *R*. When the value of *a* approaches zero, the model depicts an increasingly linear strain regime through depth (e.g., Figure 3a); conversely at nonzero values, the model reflects quadratic behavior (e.g., Figure 3b).

Lastly, the depth‐dependent derivative of equation (10) will generate the vertical strain rate:

(11) $$\frac{\partial \delta_{m}}{\partial R}=2aR+b,$$

which is linear or constant depending on the value of *a*. In the nonlinear case, it also informs the polarity point of strain reversal (i.e., the depth at which strain thinning switches to strain thickening and vice versa) when equation (11) equals 0.

Using equation (11), we model the strain regime of the ice column to 20–590 m for deployments 14a and 14b and to 20–620 m for deployment 15 (Table 1). From this, the total strain rate (TSR) can be determined by integrating over this section of the ice column and scaled according to the respective depth ranges. Note that the TSR is distinct from the rate of ice thickness change because the range limits imposed on the model do not account for melt effects occurring at the upper and lower boundaries of the ice column.

The ApRES range error from phase noise increases with increasing depth, due to progressive attenuation in the received signal power with increasing depth. Therefore, to address the heteroskedastic behavior in range, observations were weighted (Holland & Welsch, 1977) using the inverse of the measurement variance of each of the internal reflector's daily vertical velocity as a weighting factor (equation (12) and Figure 3):

(12) $$w_{n}=\frac{1}{{\sigma_{n}}^{2}},$$

where *σ*_*n*_ is the standard deviation of each measured segment *n*. This ensures that prominent internal layers influence the fitted curve more than weak internal layers.

To remove the effects of surface ablation and occasional delays in signal transmission (Figure S2), all profile pairs were shifted vertically prior to modeling the full‐column vertical strain but after calculating the daily vertical velocity profiles. This process was done using the same procedures to determine internal reflector displacement (equation (5)), but instead of 5‐m segments, we used a large section of the profile with consistently high cross correlation values at a shallow depth (40–100 m). Using a weighted robust linear regression, we determined the overall delay by extrapolating the regression to the ice surface and standardized the initial depth of all profiles to 0 m by applying an appropriate offset to the resulting displacement profile to negate the measured delay.

Noisy chirps below a certain cross‐correlation coherence threshold, which represent poor tracking of internal reflectors between measurements, were removed, causing gaps and missing data in the time series (red dotted line, Figure S4). The processed layer vertical velocity profiles were subsequently averaged into daily bins through weighted means, using the standard error of each internal reflector's measurement as the weighting factor (equation (12)). In doing so, we also removed potential temperature biases and any transient environmental changes that affect the signal propagation speed within the ApRES unit from sampling at different times throughout the day. Further information on the temperature profile of Store Glacier as well as the influence of these processes on the measured vertical ice column are detailed in supporting information S1 (Cuffey & Paterson, 2010; Doyle et al., 2018; Evans, 1965; Fujita et al., 2000; Kendrick et al., 2018; Pettersson et al., 2007; Rahman, 2016).

#### 3.2.3 Basal Topography

Using equations (1) and (2), the total ice thickness at any point in time can be determined by setting the coarse range bin center (*n*) to the basal reflector (thick dashed line, Figures 2b and 2c). The bed elevation can then be determined from subtracting the ice thickness from (i) GPS measurements of surface elevation, or (ii) a digital elevation model of surface elevation (Greenland Ice Mapping Project digital elevation model; Howat et al., 2014). Repeating this procedure for all bursts (Table 1) produces profiles of basal topography for each radar deployment. Within this paper, we show a preference for (i) and substitute the digital elevation model in place of surface GPS measurements when there were gaps in the time series.

### 3.3 GPS Measurements of Surface Ice Motion

Surface elevation and velocities (*u*_*s*_) were measured during 2014 and 2015 using dual‐frequency GPS receivers (Trimble 5700 and R7) installed on 4.9‐m‐long poles drilled 3.9 m into the ice surface. The GPS receivers were powered by a 50–100 Ah battery, solar panels, and a wind generator and sampled at a 10‐s interval. Data from the receivers were processed kinematically (King, 2004) using Track v. 1.28 (Chen, 1998) relative to bedrock‐mounted reference receivers using precise ephemeris from the International GNSS Service (Dow et al., 2009) and IONEX maps of the ionosphere (Schaer et al., 1998). Reference GPS receivers were located near the glacier terminus (STNN; Trimble NetRS; Figure 1a) and at Qaarsut (QAAR). Preference was given to STNN over QAAR due to its 30‐km baseline length compared to QAAR's 105 km.

### 3.4 Borehole Measurements of Basal Conditions

During July and August 2014, four boreholes were drilled by hot water to the bed at 605–615‐m depth and instrumented with several englacial and subglacial sensors at site S30, where the ApRES units were deployed (Figure 1b; Doyle et al., 2018). Of the data sets obtained from instrumentation of boreholes, we investigate herein the time series of tilt sensor readings to obtain a 2‐month‐long record of basal velocity. The basal velocity time series were derived by subtracting the difference between surface velocity measured by GPS and horizontal deformation rates calculated from the installed tilt sensors.

Tilt sensor time series data were processed assuming all angle variations are in the direction of flow and occur solely due to vertical variations in horizontal shear (Keller & Blatter, 2012). This method determines the vertical gradients of horizontal velocity (*∂u*_*x*_/*∂z*), where *u*_*x*_ is taken along the direction of flow. We interpolated through these sparse measurements using a temperature‐dependent flow model (Cuffey & Paterson, 2010) at specific depth points (Doyle et al., 2018).

We integrated (*∂u*_*x*_/*∂z*) cumulatively over the entire ice column to calculate the deformational velocity time series *u*_*d*_ for the period 04 August to 25 September 2014 when the tilt sensor string was operational. Subtracting *u*_*d*_ from the time‐varying surface velocity *u*_*s*_ (section 3.3) generates a time series of basal motion *u*_*b*_:

(13) $$u_{b}=u_{s}-u_{d}.$$

To match the time steps of other measurements in this study, the resulting time series was filtered with a two‐pole, low‐pass Butterworth filter with a 72‐hr cutoff period and then binned into daily averages.

The basal traction *τ*_*b*_ time series was calculated using the Mohr‐Coulomb failure criterion that describes the plastic shear strength of the porous layer that underlies Store Glacier (Hofstede et al., 2018) and several other glaciers in Greenland (Booth et al., 2012; Bougamont et al., 2014; Christianson et al., 2014; Dow et al., 2013; Kulessa et al., 2017; Walter et al., 2014). The Mohr‐Coulomb failure criterion is expressed as (equation 12 of Christoffersen & Tulaczyk, 2003):

(14) $$\tau_{b}=c_{0}+N\tan\mu ,$$

where *c*_0_ is the apparent cohesion, *N* = *p*_*i*_ − *p*_*w*_ the effective normal stress (the pressure at the ice‐bed interface), and *μ* the sediment internal friction angle. *c*_0_ can be assumed to be negligible for deforming till due to the low clay content (Cuffey & Paterson, 2010). The internal friction angle is known to exhibit little variation between glaciers (Murray, 1997). Accordingly, values of *c*_0_ (0) and *μ* (30°) were set to those of Trapridge till (Clarke, 1987), following Bougamont et al. (2014). The effective normal stress is calculated as *N* = *ρ*_*i*_ *gD* − *p*_*w*_, where *ρ*_*i*_ is the ice density, *g* the gravity, and *D* the ice thickness. Here *D* was calculated from ApRES measurements to the basal reflector, and *p*_*w*_ was recorded empirically by two vibrating‐wire piezometers installed at the ice‐sediment interface (Doyle et al., 2018).

## 4 Results

Three separate deployments over a period of 2 years report vertical velocities of internal layers during spring (deployment 14a), summer and autumn (deployment 14b), and a whole year (deployment 15). The vertical velocities varied substantially with depth and over time at and between each site (Figure 4). Within each deployment, barring occasional erroneous records affecting the entire measurement of the ice column for short (1–3‐day) periods, vertical velocities were relatively constant in error (Figure S5). During the summer and leading into the winter months, vertical velocities were largely positive in all three profiles, with more strongly positive vertical velocities measured at depth (e.g., July through December). During the early spring, vertical velocities become increasingly negative through time (January through June; Figures 4a and 4c). Where the profiles overlap in time (deployments 14b and 15 in August to December), the data appear similar with positive vertical velocities through the temporal window (Figures 4b and 4c).

While the upper 400 m of the measured ice column during the Spring of 2014 at deployment 14a revealed negative vertical velocities, where internal reflectors move increasingly closer to the antennas located at the ice surface (Figure 4a), deployments 14b and 15 during the summers and autumns (July–December) of 2014 and 2015, respectively, record positive vertical velocities, where internal reflectors move in the opposite direction away from the ice surface (Figures 4b and 4c). As well as exhibiting a change in sign with depth within a single profile for extended periods, as observed in deployments 14a (06 May to 22 June; Figure 4a) and 15 (January/February–May 2016; Figure 4c), the vertical velocities of internal layers also underwent temporal evolution within the same deployments. In the former (deployment 14a), negative vertical velocities observed in the first month and a half became increasingly positive between mid‐June and mid‐July of 2014, with the movement of deeper individual reflectors away from the ice surface occurring earlier in time than those located at shallower portions of the ice column (Figure 4a). The opposite trend was observed in deployment 15 from January 2016 onward, where shallow internal layers begin to show movement toward the ice surface earlier in time than those at depth.

When translated into vertical strain rates, the resulting profiles for all three deployments show similar variations through time and, occasionally, through depth, underlain by an overall thickening strain regime as given by consistently positive TSR values (Figure 5). Between August to February, relatively constant vertical strain thickening was observed (Figures 5b and 5c); from March through July, the vertical profile displayed increasingly complex strain behavior, where strain thinning in the upper section of ice is counteracted by strain thickening at depth (Figures 5a and 5c). We first observed such complex strain behavior within the ice column in May 2014, where the overall thickness of the thinning upper ice section increased progressively from May to late June (Figure 5a) when the point of zero strain changed from 200 to 400 m below surface. This behavior changed abruptly on 18 June, when both the rate and the proportion of the ice column exhibiting vertical strain thickening in the lower part of the glacier instead started to increase. This transition ultimately resulted in most (∼70%) of the ice column showing high rates of strain thickening by mid‐July of 2014, increasing the overall strain rate over the measured ice column. This strain thickening regime carried over and dominated deployment 14b (Figure 5b), which ceased on 04 December 2014 when the antenna array was damaged by strong winds (Table 1). During this period (leading up to 04 December), vertical strain rates showed consistent and uniform strain thickening through the ice column with rates averaging 0.0165 a^−1^. Occasionally, ephemeral bursts of sudden vertical strain thinning in the upper ice column with each lasting up to 1 day (black stars, Figure 5b) were also observed through the summer and autumn season, with most showing high (*R* ^2^ > 0.7) correlation values. Although the redeployment of the ApRES in July 2015 (deployment 15) did not show the same trend in near‐surface thinning observed the previous year in the deployment 14a time series, it confirmed that strain thickening again dominated the ice column from July to December for the second year in a row (Figure 5c). The deployment 15 time series furthermore confirmed that the vertical strain regime subsequently returned to one where thinning encroaches the upper portion the ice column, as observed at the beginning of deployment 14a. This transition was gradual and took place between March and June (Figure 5c), whereas the transition from thinning to thickening during June/July 2014 was much more rapid and lasted only a few weeks (Figure 5a).

**Figure 6:**
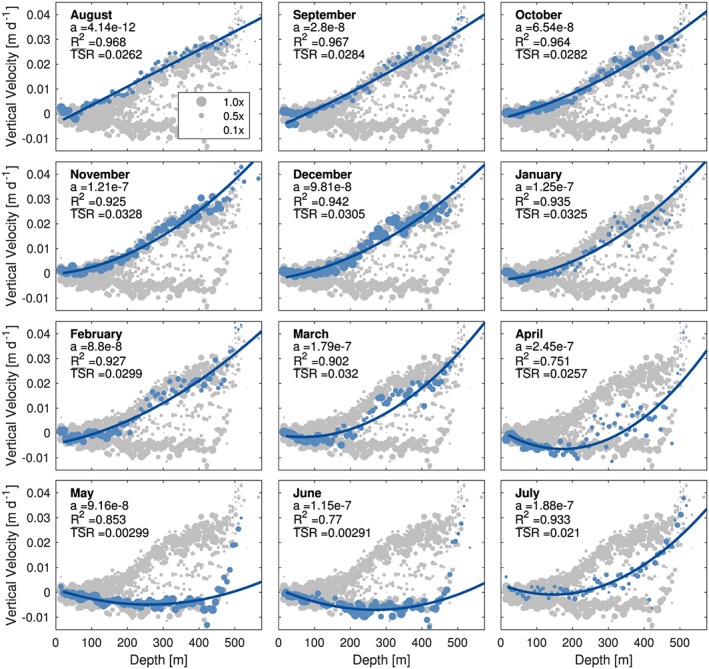
Time evolution of monthly averaged vertical velocity profiles. Marker size represents the relative weight of each reflector toward robust fitting, where larger markers carry more weight than smaller markers. The May, June, and July profiles (last row) were obtained from deployment 14a; the rest of the profiles (first three rows) were obtained from deployment 15. For each month, the curvature a (units m·m^−2^·a^−1^), the goodness‐of‐fit R ^2^ (unitless), and the $\bar{TSR}$ (units a^−1^) are displayed. The markers for the specified month in each plot is shown in blue; with the background composed of data collated from all deployments. TSR = total strain rate.

Further examination of the curvature of the model fits reveals seasonal patterns and trends in strain within the ice column as it evolves through time. During the late summer (August–October), the curvature of the strain profile *a* is positive but small (4 × 10^−12^ to 3 × 10^−8^ m·m^−2^·a^−1^), suggesting almost‐linear strain thickening along the ice column (Figure 6). Indeed, this was found in the majority of the data acquired during late summer and autumn in 2014 (deployment 14b; Figure 5b) as well as during the same seasons in 2015 (deployment 15; Figure 5c). After this period, *a* gradually increases with time up to 1 × 10^−7^ m·m^−2^·a^−1^, reflecting increasing vertical strain with increasing depth. This trend in curvature eventually introduces strain thinning near the ice surface at the peak of midwinter in January/February, when the identified point of the polarity reversal (where the strain regime switches from strain thinning to thickening) begins to descend in depth through the rest of the winter and into spring (Figure 5c). The descent of the polarity point is furthermore reflected by the decreasing values of the averaged daily total vertical strain rate ( $\bar{TSR}$) through successive months, reaching close to 0 during May and June. Only from mid‐June does the vertical velocity profile revert back to a purely thickening ice column through the course of the summer season, as evidenced by the increase in $\bar{TSR}$ values (Figure 6).

While the quadratic models of vertical strain provided a high goodness‐of‐fit (*R* ^2^ > 0.90) outside the melt season, they were unable to capture fully the nonlinearity in strain behavior during late spring and early summer, as evidenced by the markedly lower *R* ^2^ values through this period (Figure 6). This deviation was especially apparent in mid‐June, when the directional switch of the polarity point upward through the ice column caused difficulty with the quality of the model fitting (Figure 5a). At this point in time, the vertical displacement of internal reflectors deviated away from the prescribed quadratic function within deeper sections of the ice column (>420 m). Instead, the profile resembles a piecewise linear function, with slight strain thinning above 420 m, and much more substantial strain thickening below this depth (Figure 6).

## 5 What Drives Variations in Vertical Strain?

Variations in the vertical strain rate within an ice column are often attributed to spatial and temporal variations in processes occurring at the base of the glacier. These can consist of changes in (i) basal topography, (ii) basal mass balance, influenced by the sediment and water content of basal ice, coupled with the presence and pressure of meltwater at the ice/sediment interface, and (iii) basal traction (Aschwanden et al., 2012; Hindmarsh et al., 2006; Holschuh et al., 2017; Panton & Karlsson, 2015; Rippin et al., 2005; Ryser, Lüthi, Andrews, Catania, et al., 2014; Weertman, 1964; Wolovick & Creyts, 2016). Such stress variability can be generated either locally or transferred from farther afield by longitudinal and lateral couplings. There are therefore at least three scenarios with the capacity to drive the spatiotemporal patterns in vertical strain that we measured at Store Glacier:

(a) local variations in topographic setting;
(b) local variations in basal traction; and
(c) far‐field variations in glacier dynamics.

We therefore supplement the ApRES data with local observations of GPS‐ and satellite‐derived topographies and velocities in an attempt to partition the effects of these processes on the measured strain field. To this end, we discuss each scenario in turn below.

### 5.1 Local Variations in Topography

Topographically, S30 (30.3 km from the glacier terminus) is located over a local 3‐km‐wide depression within a regional bedrock high (Figure 1c). This regional bedrock high is composed of three local maxima located 28.8, 32.4, and 35.6 km from the terminus, the last maximum descending 150 m into this depression. Together, they represent a bottleneck with respect to the orientation of glacier flow. Beyond the first subglacial peak 1.5 km downstream of S30, the glacier flows over a 70‐m‐high subglacial cliff, after which it enters a deep trough that extends to depths of ∼600 m below sea level (Figure 1c).

**Figure 7:**
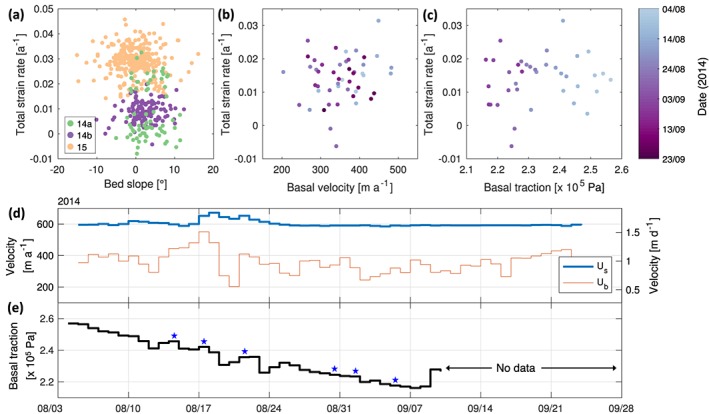
Scatterplot correlation comparing (a) total‐column vertical strain of modeled profiles in all deployments with the underlying bed slope; (b) total‐column vertical strain of modeled profiles in deployment 14b and basal velocity; and (c) total‐column vertical strain of modeled profiles in deployment 14b and basal traction. (d) Time series of surface and basal velocities inferred from kinematic GPS deployments and borehole‐installed inclinometers at S30. (e) Time series of basal traction. Blue stars show plausible instances of enhanced basal slip coincident with ephemeral instances of anomalous strain behavior (Figure 5b).

Examination of the surrounding basal topography show that ice is flowing over a local depression (Figure 1c). The eastern extent of this local depression is captured by deployments 14a and 14b, which show that the glacier at those sites flows downhill (Figures 5a and 5b). Deployment 15, which was operational for longer and therefore profiled a greater distance, showed that the glacier flowed 50 m downhill for approximately 1 month, after which it traversed relatively flat terrain before flowing 150 m uphill during the last 3 months of its operation (Figure 5c). Although the transition from vertical thickening to vertical thinning during this 3‐month period was spatially coincident with the glacier accelerating across a bedrock ridge (Figure 5c), this interpretation cannot explain why a similar strain regime was also observed in deployment 14a, which acquired data when the glacier flowed into a depression. This interpretation also cannot explain why the strain regime of deployment 14b, which also flowed downhill, recorded an altogether different strain regime. A comparison between daily TSRs and underlying bed slope, which was acquired in high spatial and temporal resolution with the ApRES system, further revealed no obvious relationship (*R* ^2^ = 0.045; Figure 7a). Therefore, given the available data, local variations in bed topography cannot alone explain the measured variations in the vertical velocities of internal layers.

### 5.2 Local Variations in Basal Traction

Basal conditions at S30 have been examined through seismic analysis by Hofstede et al. (2018), who observed the ice to be generally underlain by ∼45 m of unconsolidated sediment. However, at a smaller (∼100‐m) scale within our study area, there exists spatial variation in the observed polarity of the ice‐bed reflection (e.g., Figure 1b), suggesting that these heterogeneous patches of basal slipperiness are associated with variable amounts of water and sediment properties. We are also fortunate to have detailed information about basal and englacial conditions through instrumented boreholes drilled contemporaneously in close vicinity (∼30 m west) to deployment 14b in July 2014 (Figure 1b), where Doyle et al. (2018) reported subglacial water pressures persistently close to overburden (93–95% of *p*_*i*_). Together with GPS measurements at the ice surface, the ice velocity exhibits moderate seasonal changes at S30, where small variations in basal water pressure were concomitant with large fluctuations in surface ice velocity and uplift. These observations suggest an inefficient basal hydrological system with water flowing both at the ice‐sediment interface and within the basal sediment layer itself, resulting in a situation where basal motion at S30 is sensitive to the influx of surface meltwater to the subglacial environment.

Discrete ice flow acceleration events occurred during the melt season, when the influx of surface meltwater to the subglacial environment generated fluctuations in surface ice velocities and surface uplift (Doyle et al., 2018). This induced high variability in the coupling between basal and surface motion, for example, as seen during a recorded rainfall event between 17 and 20 August 2014 when basal motion accounted for as much as 82% and as little as 32% of surface motion (Figure 7d). When compared to the record of full‐column total vertical strain measured from ApRES, however, the contemporaneous record of basal velocity did not produce any statistically significant correlation (*p* = 0.360; Figure 7b). Similarly, the comparison between total vertical strain and basal traction (calculated from measurements of subglacial water pressure) produced no obvious correlation (*p* = 0.430; Figure 7c).

Although the time series of basal traction shows a consistent decrease through time, which Doyle et al. (2018) suggested reflects the progressive isolation of the subglacial hydrological system from influent water (Figure 7d), the strain profile of the ice column measured concurrently shows little temporal variation in either curvature or magnitude (Figure 5b). However, short periods of anomalous strain behavior, each only lasting up to a day (blue stars, Figure 5b), are observed to be temporally coincident with instances of enhanced basal slip (blue stars, Figure 7e). Similar stick‐slip behavior has previously been observed by Ryser, Lüthi, Andrews, Catania, et al. (2014) in data obtained from borehole‐installed tilt sensors, where diurnal variations in tilt were correlated with water pressure at a time lag with increasing depth from the ice surface—that is, sensors closer to the surface reacted earlier than those in the vicinity of the bed. These covariations were explained by sudden changes in basal slipperiness, resulting in local “caterpillar‐like” ice motion. Within our data, the short (≤1 day) bursts of vertical strain thinning in the upper half of the ice column could be seen to react earliest and most prominently in response to the changes in basal traction, before quickly reverting back to the overarching strain thickening regime.

### 5.3 Far‐Field Variations in Glacier Dynamics

**Figure 8:**
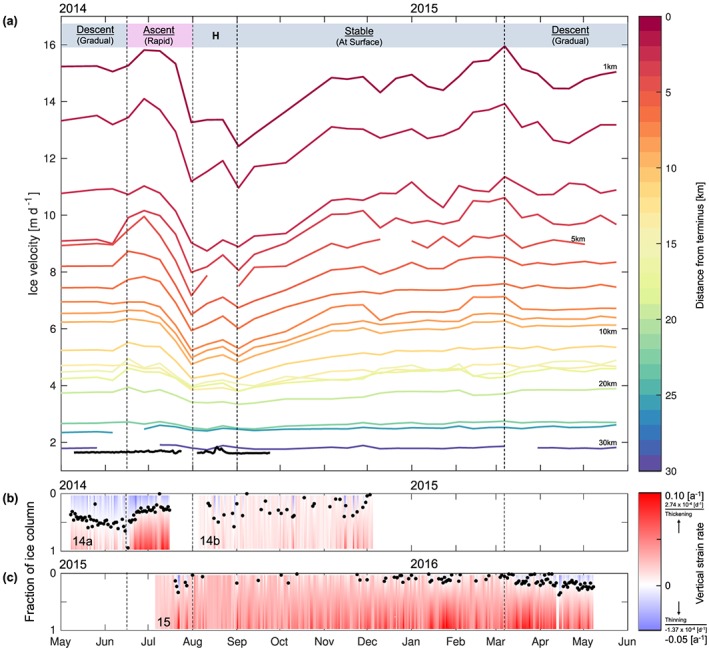
(a) Time series of surface velocity sampled every 1 km along the central flowline of Store Glacier (Figure 1a), extracted from sequential TerraSAR‐X imagery obtained between 2014 and 2015. The surface velocity at S30 (30.3 km from the glacier terminus) is shown in thick black. (b) Time series of the location of polarity switch between vertical thinning (blue) and thickening (red, Figure 5) as a fraction of the total ice column thickness for deployments 14a and 14b (May–December 2014). (c) Same as (b) but for deployment 15 (July 2015 to May 2016). The duration of seasonal phases of the movement of strain polarity are marked in (a) as either descending down (blue) or ascending up (pink) the ice column. Within the latter, the observations of surface meltwater influences on the flow velocity are superimposed over the summer slowdown (“H”).

In fast‐flowing glaciers, flow variations initiated near the terminus have been found to affect flow farther inland through longitudinal coupling, where perturbations can be transmitted over horizontal distances extending up to 10× to 20× the local ice thickness away from the region where the change in basal conditions occurred (Kamb & Echelmeyer, 1986; Price et al., 2008). To examine this effect, we examined the upstream propagation of ice flow variations initially occurring at the glacier terminus through analyzing contemporaneous TerraSAR‐X surface velocity data sets (Figure 8a). These data sets, which cover up to 30 km from the glacier terminus, encompass the fastest flowing portion of Store Glacier, including our field site, S30. The satellite velocity data, acquired from May 2014 to June 2015 at a temporal resolution of 11 days, show a distinct seasonal pattern of ice velocity. This variation is especially conspicuous near the calving terminus where surface motion, following a short‐lived increase in velocities in June 2014 to a high of 15.8 m/day, decelerated rapidly thereafter to reach an annual minimum (12.4 m/day) in late July 2014. Afterward, surface velocity increased gradually through the winter and spring to peak once again, averaging 15 m/day between March and June 2015 (Figure 8a).

**Figure 9:**
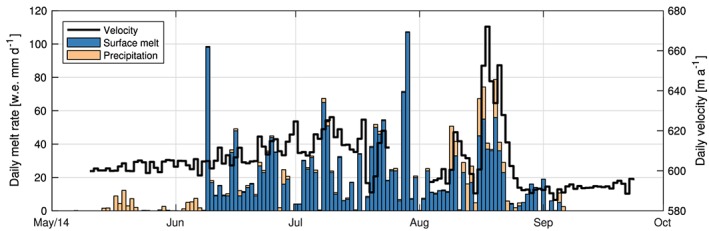
Time series through 2014 of surface velocity, surface melt rate, and local precipitation at S30. A 10‐day gap is present in the GPS time series between 24 July and 04 August due to a disrupted power supply.

These seasonal variations are observed to propagate at least 30 km up‐glacier from the terminus. Although minor compared to the absolute velocity, the pronounced deceleration commences during the latter half of June 2014 (Figure 8a) and coincides with the onset of summer melt on 09 June (Figure 9). This phenomenon is thought to not be caused by calving processes alone (Todd et al., 2018). Instead, we hypothesize that this decrease in ice flow, which is a persistent characteristic of Store Glacier (Ahlstrøm et al., 2013; Howat et al., 2010), is most likely a glaciological response to the sudden injection of vast amounts of surface meltwater to the bed of the ice sheet. Although this response is similar to those inferred along the land‐terminating ice margin (Bartholomew et al., 2010; Chandler et al., 2013; Van De Wal et al., 2015), our local observations of pressurized meltwater within an inefficient subglacial drainage system, coupled with remote sensing measurements of glacier‐wide variations in ice flow velocities, are akin to the processes underlying fast‐flowing marine terminating glaciers (Lüthi et al., 2002; Meier et al., 1994), as well as glaciers in surge (Kamb et al., 1985). At Store Glacier, the buildup of ice volume over the quiescent winter period is suddenly released by the concurrent breakup of the proglacial ice mélange, removing a vital source of stabilizing backstress (Toberg et al., 2016). The initial acceleration in flow is thought to be driven by enhanced submarine melting and calving at the glacier front (Morlighem et al., 2016), as well as the presence of widespread pressurized basal water residing in small cavities within an inefficient subglacial drainage system (Doyle et al., 2018). As discharge reaching the small cavities at the ice‐bed interface becomes critically high, turbulent heat dissipation from the incoming water flux encourages conduit growth and allows for greater discharge fluxes and yet more energy dissipation (Kamb, 1987). With meltwater increasingly captured by the growing channels and with water pressure falling in those channels, frictional resistance along the bed increases, and the glacier eventually experiences a slowdown in flow (Kamb, 1987). Outside the melt season, the gradual increase in satellite‐observed ice flow during the autumn and winter from 12.4 m/day in August 2014 to the mean maximum of around 15 m/day in March 2015 is consistent with ice flowing gradually faster as subglacial water pressure builds up in an inefficient basal water system consisting of small cavities, which accommodates a continuous production of basal meltwater while subject to creep closure due to the weight of the ice above.

The characteristic seasonal velocity change of Store Glacier coincides with the observed seasonal variations in vertical velocity and strain reported above. From mid‐June to late July, when peak summer melting occurred (Figure 9), we infer a rapid slowdown extending from the terminus to within a few kilometers downglacier of S30. Although the slowdown is very small in our GPS record from S30 (Figure 9), we note that the longitudinal and transverse stress gradients theoretically extend well beyond the area over which basal conditions have changed. This inference is consistent with the strain regime observed within the ice column at S30 during this period, where vertical thickening at depth rapidly overtook the previously permeating vertical thinning in the upper portion of the ice (Figure 8b). As the summer melt season comes to a close, the ice velocity returns toward its balanced, winter values, causing longitudinal stress gradients to wane. Here we infer the basal hydrological system to again consist of small cavities distributed over a large area, the former which grow in size as the flow of the glacier gradually increases during winter and spring. At S30, we measure relatively constant vertical thickening until December, after which vertical thinning gradually and progressively increases in the upper part of the glacier propagating through depth, culminating with concurrent half‐column thinning and half‐column thickening in early June the following year (Figure 8c).

## 6 Discussion

The combination of field and satellite data leads us to hypothesize that longitudinal coupling provides the most likely explanation for the spatiotemporal variations observed in the vertical velocity of englacial reflectors and the vertical strain rates at S30. Our observations of vertical strain span 2 years, from May 2014 to May 2016, with the changes observed over this period having a notable seasonal appearance. With a gap in data stemming from equipment failure between December 2014 and July 2015, we can, however, only partly ascertain the seasonality through repeat measurements. While we cannot exclude the possibility that nonseasonal factors could be important, a seasonal forcing is a simpler and more likely driver of the observed variation in strain. Given that data were acquired in three separate deployments at three different times, it may be plausible that the observed trends in the strain rate time series may be influenced by the variability of transverse and shear strain components in response to seasonal changes in basal conditions and ice velocities. Small deviations between the deployment time series, such as the lack of near‐surface thinning observed in deployment 15 in July 2015 when compared to the record obtained from deployment 14a a year prior (Figures 5a and 5c), may be the result of these spatial and temporal differences. Notwithstanding, the spatial separation between data points in deployments 14a, 14b, and 15 was less than a few hundred meters, with all three deployments within a kilometer from the central flowline, both extremely small in comparison with the size of the glacier. The seasonally consistent variations captured in multiple deployments furthermore support the interpretation that the data were captured in largely the same setting. Therefore, at Store Glacier, concomitant seasonal variations in both the vertical strain regime and ice velocities are likely to be far‐field glaciological responses superimposed on localized variations in ice flow.

While most of the glacier front experienced rapid acceleration followed by dramatic deceleration at the onset of summer melt in June (Figure 8a), surface velocities at S30 instead exhibited a gradual increase through the course of the melt season. Superimposed on this dampened seasonal effect are large fluctuations in velocities, driven by injection of locally produced meltwater to the bed at S30 (Figure 9). At S30, these synchronous patterns between daily velocities and surface melt rates, combined with coincident measurements of borehole water pressure, all support a spatially and temporally homogeneous and inefficient basal hydrological system underlying the drill site (Doyle et al., 2018) that, in contrast to the channelized water systems developing closer to the ice front, remain relatively inefficient and unchanged throughout the year. In addition to the observations on Store Glacier, the inferred presence of distributed basal drainage at higher elevation is also consistent with observations on land‐terminating glaciers (Andrews et al., 2014; Hoffman et al., 2016). The longitudinal coupling effect observed here may be part of a catchment‐wide phenomenon that is governed by melt‐induced perturbations from surface water forming and reaching the bed, which propagate upglacier through the course of the summer melt season. A detailed numerical analysis of the longitudinal coupling effect by Christoffersen et al. (2018) supports the vertical thickening observed throughout the ice column starting in July at S30. Here widespread melt occurring at the surface lubricates the bed at S30, encouraging faster velocities, while ice flow at lower elevations becomes markedly slower in response to the formation of an efficient basal drainage system from large, continued meltwater influx into the subglacial environment. This regime of vertical thickening persists throughout autumn and winter (Figures 8b and 8c), which shows that it takes several months to build up high water pressure in the distributed basal drainage system forming at elevations below S30 after the summer melt season. Only in March does the ice column at S30 begin to experience vertical thinning in response to ice flow acceleration induced by the repressurization of the distribution basal drainage system at lower elevations, where an efficient system develops in summer (Figure 8c). The encroaching thinning during this period (from March to June) may be caused by the emergence of downstream longitudinally extensional forces that superimposes onto the originally thickening vertical strain regime from the autumn and winter months (August–February). Given that the temperature profile measured at the study site is indicative of fast flow, where cold ice dominates near the surface to below −20 ° C halfway down the ice column to near‐temperature conditions at the bed (Doyle et al., 2018), the downstream stresses could conceivably be accommodated mostly in the upper reaches of the ice column, where the ice is colder and stiffer.

Although S30 is located upstream from the region where basal hydrology induces large seasonal variations in ice flow (“H” in Figure 8a), our observations show that vertical strain nevertheless undergoes distinct seasonal transformations. These transformations involve (i) a gradual transition with vertical thinning expanding and extending approximately halfway through the ice column, resulting in a distinctly nonlinear vertical strain profile (March–June); (ii) a shorter period during which vertical thinning is reduced to the upper portions of the ice column, with vertical strain remaining distinctly nonlinear (June–July); and (iii) an extended period with vertical thickening throughout the full column and a linear vertical strain regime (August–March). To match these temporal variations in vertical strain, the curvature term (*a*) within the quadratic model was allowed to fluctuate (equation (10)). While the output models produced had a high goodness‐of‐fit for all deployments (*R* ^2^ > 0.75), we acknowledge that, during (i) (e.g., May and June; Figure 6), our automated model was unable to completely encapsulate the vertical velocity distribution at the deepest sections of the ice column (>420 m). Given the high strain rates observed in the lower half of the ice column during this time, increasing with depth, this observed nonlinearity in strain behavior is most likely linked to the enhanced basal deformation observed in boreholes, with approximately 70% of the deformation occurring in the lowermost 100 m of the ice column and 40% in the lowermost 50 m (Doyle et al., 2018). Supported by coincident seismic studies (Hofstede et al., 2018), this warm basal zone of enhanced deformation has been interpreted to consist of “softer,” potentially pre‐Holocene ice (Doyle et al., 2018). With an ice column consisting of Holocene as well as softer Wisconsin‐aged ice, enhanced deformation in the latter, thought to occupy the lowest 150 m of the ice column, should be expected for a glacier flowing over a relatively nondeforming strong bed. The suggestion of highly deformable ice may also explain why internal layers within these regions were difficult to track (Figure 4; Young et al., 2018). The vertical velocity profiles observed in this study are consistent with a high concentration of strain within this highly deforming layer, with colder ice higher up the ice column resembling a “stiff beam,” resisting these stresses (Ryser, Lüthi, Andrews, Catania, et al., 2014). Therefore, the compressional effect from strong longitudinal coupling observed in the summer melt season could be thought to trigger the temporally rapid transition from vertical strain thinning to thickening. This effect is seen to manifest first within the lowermost internal layers, before gradually propagating up the ice column into colder ice, which, because of its increased stiffness from low temperatures, is likely to be less prone to deformation.

The degree of influence that basal slip has on englacial ice deformation likely depends on the spatial variability in basal slipperiness imposed by the basal boundary condition. At the scale of 5–10×, the local ice thickness, both Ryser, Lüthi, Andrews, Catania, et al. (2014) and Holschuh et al. (2017) attributed variable surface velocities and ice deformation patterns in both space and depth to periodic patches of different basal slipperiness, while at a finer scale (≤1× the ice thickness), other studies (Balise & Raymond, 1985; Kamb & Echelmeyer, 1986; Mair et al., 2001) do not observe any clear relationship between basal drag and glacier motion due to horizontal coupling with surrounding areas. The strain regime in the overlying ice column may also be dependent to the degree of basal friction imposed at the ice‐bed interface. While small fluctuations in basal slipperiness have been observed to only perturb the lowermost portions of the ice column, larger, sudden changes in basal traction are thought to affect the strain regime of the entire ice column (Holschuh et al., 2017; Ryser, Lüthi, Andrews, Catania, et al., 2014), potentially generating strain regimes in this depth section antithetical to those seen at shallower depths. Although we find ephemeral “shock” instances where the strain regime of the ice column experienced anomalous behavior correlating to periodic drops in basal traction (Figure 7e), we do not find any significant correlation between variations in basal and vertical velocities (Figure 7b). Similarly, we also find variations in fine‐scale basal topography unable to explain variations seen in the vertical velocity profiles at the same spatial scale (Figure 7a). However, at sufficiently long wavelengths (5–10× the ice thickness), internal layering is expected to drape along the subglacial topography (Hindmarsh et al., 2006). Therefore, at this scale, significant subglacial obstacles can exert marked hypsometric control over the morphology of internal layers (Bingham et al., 2015).

## 7 Conclusions

We have presented time series of vertical velocity and strain in a fast‐flowing, marine‐terminating outlet glacier in West Greenland. The time series, which was derived using autonomous phase‐sensitive radio‐echo sounding to track day‐to‐day displacements of englacial layers with millimeter accuracy, provide a novel insight into the mechanics of fast glacier flow. The data, which were captured with unprecedented resolution and detail, revealed variations in vertical velocity and strain within glaciers that are complex and far more pronounced than previously reported (Cuffey & Paterson, 2010; Gudmundsson, 2002; Paterson, 1976; Sugiyama & Gudmundsson, 2003).

Although we show that variations in the vertical strain regime of the ice column are unlikely to be caused by the local setting of the glacier at site S30, where the data were collected, we observe instances where sudden drops in basal traction may have “shock” effects on the strain regime of the ice column at the daily time scale. More importantly, though, we found that the effects of hydrologically driven far‐field horizontal stress transfer are able to explain the seasonal variations seen in vertical strain. Further work investigating the causes and consequences of longitudinal stress coupling is required to conclusively determine the mechanisms that govern the local deformational regime.

By observing the corresponding patterns of radar‐derived vertical ice velocities and far‐field glacier dynamics observed in TerraSAR‐X satellite imagery, variations in vertical velocity at site S30 are thought to be primarily driven by longitudinal coupling. Furthermore, concomitant seasonal variations in both the vertical strain regime and ice velocity are likely far‐field glaciological responses superimposed on localized variations in ice flow. During the peak of the summer melt season between June and July, the rapid slowdown near the terminus of Store Glacier induced compressive longitudinal stress gradients cascading upglacier to S30, quickly replacing the vertical thinning in the upper portion of the ice with consistent vertical thickening at depth. As surface meltwater production ceases past the melt season in September, the basal hydrological system becomes increasingly inefficient, and the entire frontal region of the glacier gradually increases in speed until reaching a steady maximum velocity in March, averaging 15 m/day at the glacier terminus. Only at this time does the ice column begin to reexperience the vertical thinning in response to the change in longitudinal stress gradients, gradually offsetting the vertical thickening at depth until the onset of surface melt the following June.

The distinct response of local strain at site S30 to temporal variability of ice flow taking place tens of kilometers away indicates that the transport of meltwater along subglacial drainage paths causes flow variations from the terminus all the way to 30‐km inland, thereby connecting site S30 with the fastest flowing sections downglacier (Figure 8). From mid‐June to late July, when the basal water system accommodates the melt season's peak production of surface water, efficient channels form a network, which drains the glacier more effectively and causes a pronounced slowdown at elevations extending up to but not beyond site S30. At higher elevations, the glacier is expected to naturally flow at a slower rate (e.g., Fitzpatrick et al., 2013) and presumably is only weakly modulated by surface meltwater input due to limitations in the ability of basal channels to form (Mankoff & Tulaczyk, 2017). Ice flow at site S30 therefore becomes compressive, with thickening taking place throughout the ice column. The vertical velocity profile of the ice column here can be approximated well with a flexible quadratic model, and the goodness‐of‐fit of this model is high (*R* ^2^ > 0.90) from July to late January and early February the following year. In late February or early March, we find vertical velocity to gradually induce vertical thinning in the upper half of the ice column, while the lower half continues to thicken. At this point the curvature of the best‐fit model for the observed strain begins to increase. Eventually, in the months preceding the onset of summer melt (April–June), the two different strain regimes become so different that model fails to capture the nonlinearity of the thickening deeper section (*R* ^2^ ≤ 0.85). While we cannot fully explain this depth transition in strain regime, we infer that it is associated with a relatively thick layer of Wisconsin‐age ice present in the lowest ∼100 m of the ice column at S30 (Doyle et al., 2018; Hofstede et al., 2018). The lower degree of viscosity attributed to Wisconsin ice facilitates enhanced deformation, in contrast to the Holocene ice residing above this layer. The latter, being more viscous and cold, may thus act as a stiff beam, which accommodates the far‐field transfer of stresses via longitudinal coupling (Christoffersen et al., 2018). The latter plays a crucial role in the force balance of the ice sheet in summer but may also be important in winter and spring, when a significant increase in the water pressure inside small basal cavities reduces the basal traction, resulting in faster glacier motion. The hydrological control on ice flow indicates that Store Glacier poses similar traits to other fast‐flowing, soft‐bedded glaciers and ice streams.

## Supporting information



Supporting Information S1

Figure S1

Figure S2

Figure S3

Figure S4

Figure S5

Figure S6
