# Supplementary material for: Physical Conditions of Fast Glacier Flow: 3. Seasonally‐Evolving Ice Deformation on Store Glacier, West Greenland
Source: J Geophys Res Earth Surf. 2019 Jan 30;124(1):245–67. doi: 10.1029/2018JF004821 (PMC6472443; doi:10.1029/2018JF004821)
Supplement: Supplementary file 1 — Supporting Information S1 [file JGRF-124-245-s001.pdf]

Supporting Information for "Physical conditions of fast glacier flow: 3. Seasonally-evolving ice deformation on Store Glacier, West Greenland"

T. J. Young[1,2], P. Christoffersen[1], S. H. Doyle[3], K. W. Nicholls[2], C. L. Stewart[1], B. Hubbard[3], A. Hubbard[4], L. B. Lok[5], P. V. Brennan[6], D. I. Benn[7], A. Luckman[8], M. Bougamont[1]

[1]Scott Polar Research Institute, University of Cambridge, Lensfield Road, Cambridge CB2 1ER, United Kingdom
[2]British Antarctic Survey, National Environmental Research Council, High Cross, Madingley Road, Cambridge CB3 0ET, United Kingdom
[3]Centre for Glaciology, Department of Geography & Earth Sciences, Aberystwyth University, Aberystwyth SY23 3DB, United Kingdom
[4]Centre for Arctic Gas Hydrate, Environment and Climate, Department of Geology, the Arctic University of Norway, Tromsø, Norway
[5]Department of Engineering, Lancaster University, Gillow Ave, Bailrigg, Lancaster LA1 4YW, United Kingdom
[6]Department of Electronic & Electrical Engineering, University College London, Torrington Place, London WC1E 7JE, United Kingdom
[7]School of Geography & Sustainable Development, University of St. Andrews, St. Andrews KY16 9AL, United Kingdom
[8]Department of Geography, Swansea University, Swansea SA2 8PP, United Kingdom

Contents

1. Introduction
2. Extended methods
3. Figures S1 to S5

1 Introduction

The supporting information includes a detailed explanation of the methods used to obtain full-column profiles of internal layer vertical velocities, as well as five supplementary figures.

Corresponding author: T. J. Young, tjy22@cam.ac.uk

2 Extended Methods: ApRES Measurements of Vertical Deformation

2.1 Pre-processing Procedure

Occasionally, the noise level of an individual chirp was significantly elevated due to user error, external interference, significant variations in the temperature of the network analyzer during measurement, or some combination of these factors [*Kingslake et al.*, 2014]. Here, contaminated chirps were removed prior to phase processing following *Stewart* [2018], which uses an automated iterative process to determine outliers based on their root-mean-square (RMS) difference from the burst-mean signal.

During operation, the ApRES may experience delays in burst transmission either internally within the system or within the cables connecting the ApRES to the antennas. As two different cable lengths of 5 and 10 m were used within each array, each resulting antenna pair will use one of three different cable combinations, where pairs with longer combinations (i.e. 5 m/10 m and 10 m/10 m) exhibited a positive offset in range in the received signal (+5 or +10 m) relative to pairs using the shortest possible combination (i.e. 5 m/5 m). The cable delays were removed by shifting each chirp profile in the range domain to align the direct breakthrough with all antenna pair combinations. Computationally, this shift was achieved by applying a phase gradient in the frequency domain equivalent to the range offset generated by the additional cable lengths—essentially, an electrical delay. Mathematically, this shift was implemented by recalculating the phase offset [$-K\tau^2/2$; Eq. 13 of *Brennan et al.*, 2014] to instead equal the range offset generated by the different cable lengths.

Specifically, this is shifted in the coarse and fine range:

$$R_{c,o}(n) = \frac{\zeta_i p}{2\Delta R_c} \tag{S1}$$

$$R_{f,o} = \frac{2\pi\zeta_i f_c}{c} \tag{S2}$$

where c is the speed of light, and ζ_i is the ice-equivalent cable length proportional to the addition in physical cable length ζ_c and the dielectric permittivities of co-axial cable ($\varepsilon_c = 2.2$) and ice ($\varepsilon_r = 3.1$):

$$\zeta_i = \zeta_c\sqrt{\varepsilon_c/\varepsilon_r} \tag{S3}$$

Note that Eqs. S1 and S2 are subject to the same bounds as their counterparts (Eqs. 1 and 2), respectively.

2.2 Estimation of error

Excluding errors arising from within the ApRES unit, which are detailed in *Brennan et al.* [2014], the overall error in ApRES-measured vertical velocities and strain rates broadly arise from assumptions made in characterizing the underlying ice column and statistical variability within the phase processing procedure, which in turn can be categorized into four sources: (i) variations in the complex dielectric permittivity of ice due to temperature variations within the ice column [*Fujita et al.*, 2000]; (ii) phase noise from the internal reflector; (iii) compaction of the vertical ice column from near-surface firn, if present; and (iv) discrepancies between observed and modeled vertical strain from inappropriate model choice. Below, we examine the effects of these errors.

i. Dependence of signal propagation to relative permittivity

Being located within the ablation zone, S30 experiences drastic seasonal fluctuations in temperature ranging from −40 °C in the quiescent winter months to more than 10 °C at the peak of the summer melt season, where the presence of surface meltwater drastically alters the physical characteristics of the incident ice column. As both changes in the temperature and composition of ice drastically affects its relative permittivity, and hence the speed of signal propagation, such changes will undoubtedly contribute most to uncertainties within measured displacements of internal reflectors. While the attenuation of the radar signal will simply result in the inability to detect and track the movement of internal reflectors, particularly at depth, the velocity of the ApRES signal propagation through ice is particularly sensitive to ice temperature.

The temperature profile within the ice column is heavily influenced by englacial and basal heat sources and sinks and, for S30, varies from temperatures near the pressure melting point just above the ice-bed interface to -21.24 ± 0.05 °C near the centre of the ice column [*Doyle et al.*, 2018]. As such, the signal propagation speed through the vertical ice column at S30 is highly heterogeneous. This would present a significant issue if the radar was used to quantify absolute ice thickness to high precision and did not account for these temperature variations. The velocity of the radar signal in ice is dictated by the relative dielectric

permittivity of the medium, and is often assumed to be between 3.10–3.18 (equivalent to 168–171 m μs^{-1}), with lower velocity values for lower ice temperatures [*Fujita et al.*, 2000]. Within this thesis, we use a relative dielectric permittivity value of $\varepsilon_r = 3.10$, which corresponds to wave propagation speeds in cold (<200 K) ice. Resulting output values are therefore likely to overestimate the absolute ice thickness: given a nominal range of 600 m using $\varepsilon_r = 3.10$, increasing ε_r to 3.18 will result in a 7.70 m decrease in ice thickness. Although we do measure absolute ice thickness within this chapter, its primary purpose is to show relative variations in basal topography along the flowline.

Similarly, temporal fluctuations in temperature within the ice column will also have a pronounced impact on signal wave propagation (~23 000 m s^{-1} K^{-1}). Specifically, there is a ~9 mm change over 600 m given a temperature increase in 0.1 °C. As variations in englacial ice temperature occur over timescales of decades and longer [*Pettersson et al.*, 2007; *Cuffey and Paterson*, 2010], and as we are measuring relative change instead of absolute thickness, we consider this source of error to be very small. Nevertheless, any anomalous movement in internal layers would have been already assumed into the total observed depth of each internal reflector and their errors accumulated through daily averaging, with larger errors accounted for through weighting (Eq. 12) and with hourly and daily outliers removed altogether from model fitting (Fig. S4).

Fluctuations in the ambient air temperature immediately surrounding the ApRES may also delay the breakthrough from the transmitting antenna, where warmer temperatures increase the signal propagation speed. This can be avoided by burying the ApRES in snow to provide insulation against temperature fluctuations, by restricting samples to the same time each day, or by incorporating the temperature effect into the cable delay. Because the ApRES arrays were installed directly on the ice surface in the ablation area, the first option can be excluded, and therefore we address this issue through the latter two options, with each burst pair f and g separated by a period of 24 h. In general, observations both from laboratory and field experiments conclude that the influence of temperature on internal reflector range detection is manifested as a simple time delay [*Rahman*, 2016].

Lastly, transient environmental changes, such as the generation of a firn aquifer from summer meltwater, are likely to affect the dielectric properties of the upper portions of the ice column. Recent studies using the same ApRES datasets have estimated the firn aquifer to extend down to 41 m below the ice surface, impounding substantial surface meltwater beyond

the summer melt season into winter, when it is either released or refrozen [*Kendrick et al.*, submitted]. Asides from markedly attenuating the received signal, the seasonal presence of meltwater will increase the signal velocity within the affected section of ice [Eq. 7 of *Fujita et al.*, 2000]. This is primarily due to the differences in the dielectric permittivities between pure water and pure ice [approximately ε_w = 80 and ε_r = 3.10, respectively; *Evans*, 1965]. Given a sudden increase in the water content, internal layers within this zone would be detected at increasingly shallower depths, causing the entire zone to falsely experience heightened strain thinning, with layers falling below the lower boundary experiencing a negative bulk offset. Accordingly, the upper boundary of the identification and tracking of internal reflectors has been set to 40 m, and any bulk offset in internal layers (i.e. the parameter c in Eq. 10) removed through taking the derivative to obtain the vertical strain rate.

ii. Phase noise from internal reflectors

Following *Rosen et al.* [2000] and *Stewart* [2018], phase noise from a specified internal reflector (σ_n) was calculated from the maximum coherence between two sequential cross-correlated bursts:

$$\sigma_n^2 = \frac{1}{2N_L}\frac{1-\hat{\gamma}_{fg}\left(n,\hat{\Lambda}\right)^2}{\hat{\gamma}_{fg}\left(n,\hat{\Lambda}\right)^2} \tag{S4}$$

Here, N_L is the number of elements within the segment n. These estimates of error were then used as weights for robust curve fitting to obtain modeled vertical strain rates (Eq. 12).

iii. Firn compaction

As ApRES arrays were deployed directly on the ablating ice surface, snow and firn beneath the antennae do not contribute towards error in this study. The downward displacement of the ApRES caused by ablation was taken into consideration in the model fitting process, as described above.

iv. Discrepancies between observed and modeled vertical strain

The statistically automated selection of models to fit the depth profiles of vertical velocity gave high significance for the large majority of the time series (Fig. 5). The quadratic

model used approximately characterize variations in the shape of the velocity profiles, but occasionally fails to capture trends in the movement of internal reflectors at depth (>$^2/_3$ of the total ice thickness). Furthermore, we were unable to consistently identify and track the movement of the deepest internal reflectors (Fig. S4), and as a result, had to extrapolate through this range to yield full-depth vertical velocities. Although at present there exist no known studies, modeled or observed, that suggest a polarity reversal of vertical movement or deformation in this lowest section of the ice column, we cannot definitely preclude the possibility of deformation within this section that deviates from the model extrapolation.

2.3 Quality assessment

Because the vertical displacement of internal reflectors were assessed through cross-correlation of short segments of paired profiles, the accuracy of a reflector's determined vertical displacement wholly depends on the coherence of the correlation. In general, the coherence of the cross-correlation should decrease with increasing depth due to the englacial attenuation of the transmitted signal.

Occasionally, burst pairs produce either very few measurements of vertical displacement or undulating profiles with very steep velocity gradients inconsistent with neighboring displacement profiles in time. These erroneous measurements were found to be either coincident with environmental influences (e.g. pooling of surface meltwater near the radar) or some form of instrument malfunction, both of which greatly reduced the correlation strength when internal reflectors were matched. The low coherence of numerous internal reflectors within a single burst pair resulted in a low usable fraction of segments useful for fine-scale analysis. In these instances, the entire burst pair was automatically excluded if the fraction of segments through the entire ice column exceeding a specified minimum amplitude correlation did not meet a specified fractional threshold (dashed red; Fig. S4). Removal of an entire burst pair, as a result, presented occasional gaps in the time series.

Within the three deployments, the instrument operated either in MIMO, where 64 chirps were cycled through the combinations of virtual transmitting and receiving antenna pairs within one burst, or in a quasi-monostatic mode, where all 64 chirps were transmitted and received between one transmitting and one receiving antenna. While deployments 14a and 14b operated solely in MIMO, deployment 15 alternated between MIMO and a quasi-monostatic operation (Table 1). Because all chirps in deployment 15 were stacked upon one

virtual antenna, rather than interspersed between the gridded locations of the synthetic aperture, the phase noise is reduced and a higher threshold was used (Fig. S4c). On the other hand, due to the data quality being hampered by intense surface melt in August of 2014, a lower threshold was implemented to reduce gaps in the time series of deployment 14b (Fig. S4b).

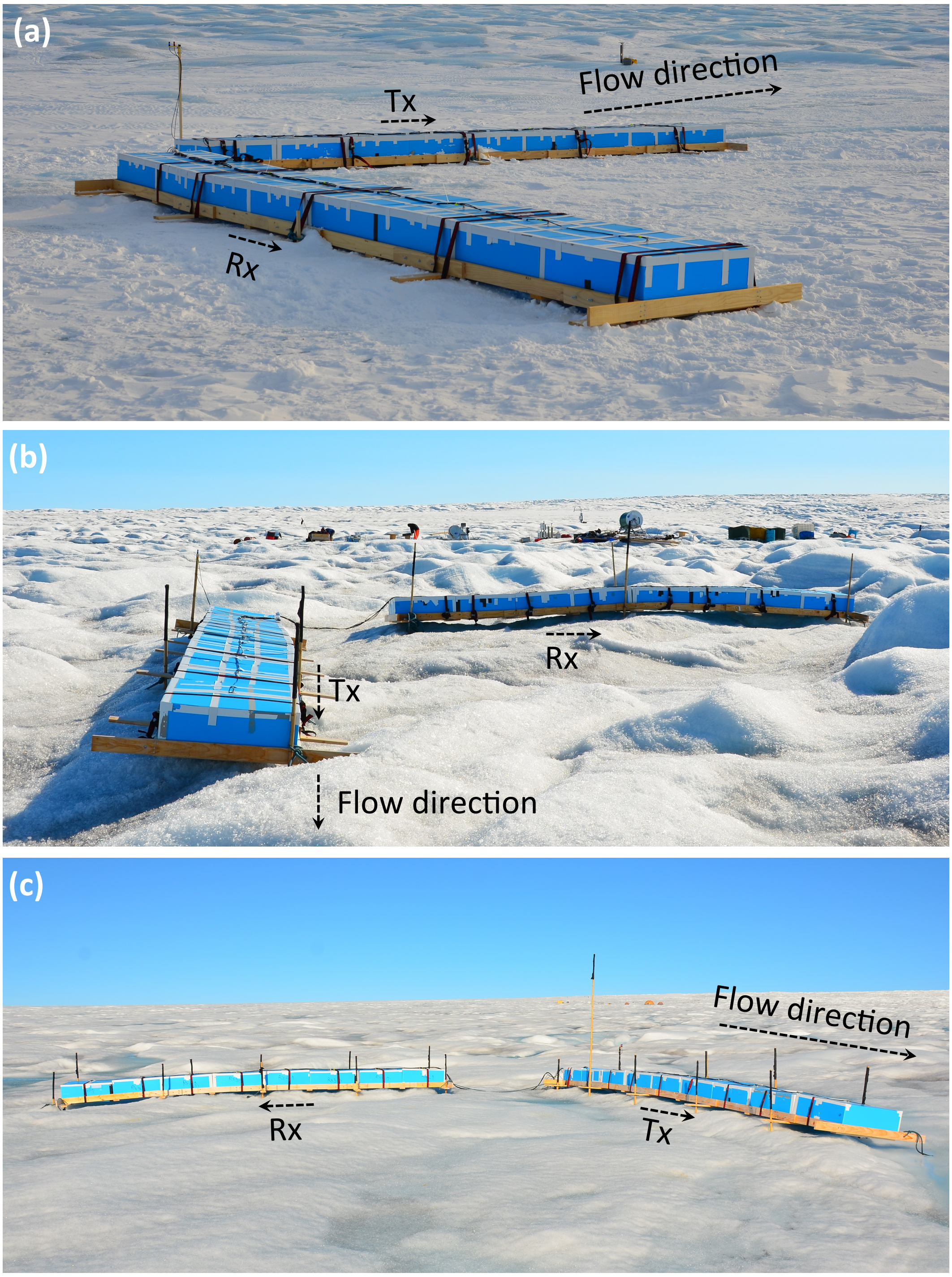

174 **Figure S1.** Setup configurations of ApRES array deployments (a) 14a; (b) 14b; and (c) 15. The principal
175 flow direction is oriented west-southwest (262°).

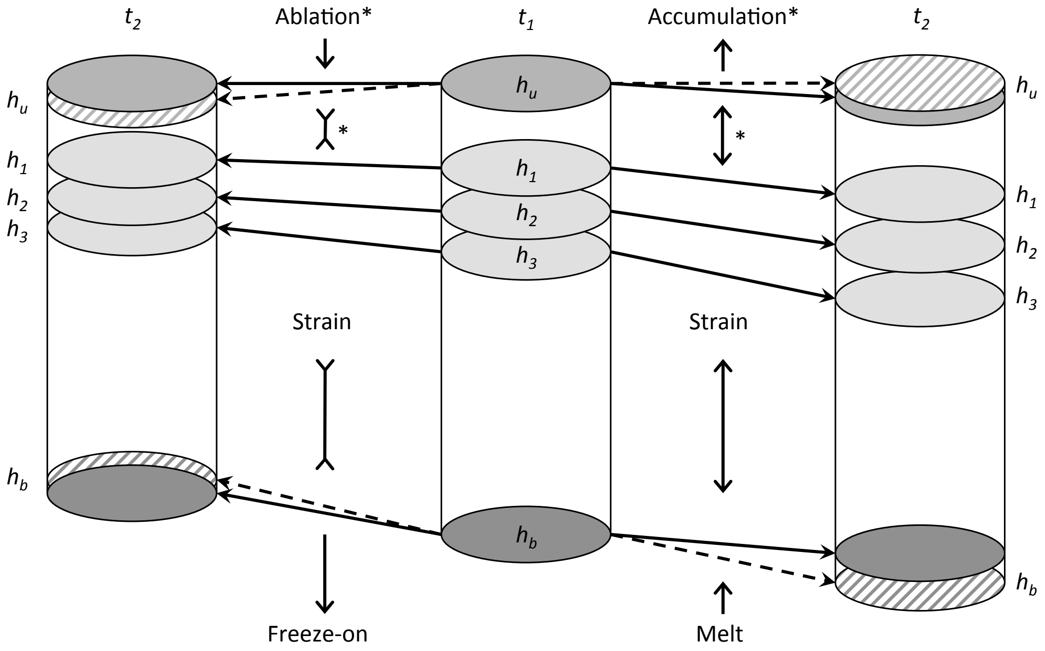

176 **Figure S2.** Schematic picture of the mass conservation of an ice column at two discrete times t_1 and t_2. Internal layers h_x, where x represents positive integers, are tracked through time to determine vertical displacement, and their relative movement to each other determines vertical strain. Changes in the upper and lower surfaces h_u and h_b are measured relative to internal layers. Detection of any changes in the upper surface also incorporate internal delays in signal transmission within the radar unit, if present (marked by an asterisk). Adapted from *Jenkins et al.* [2006].

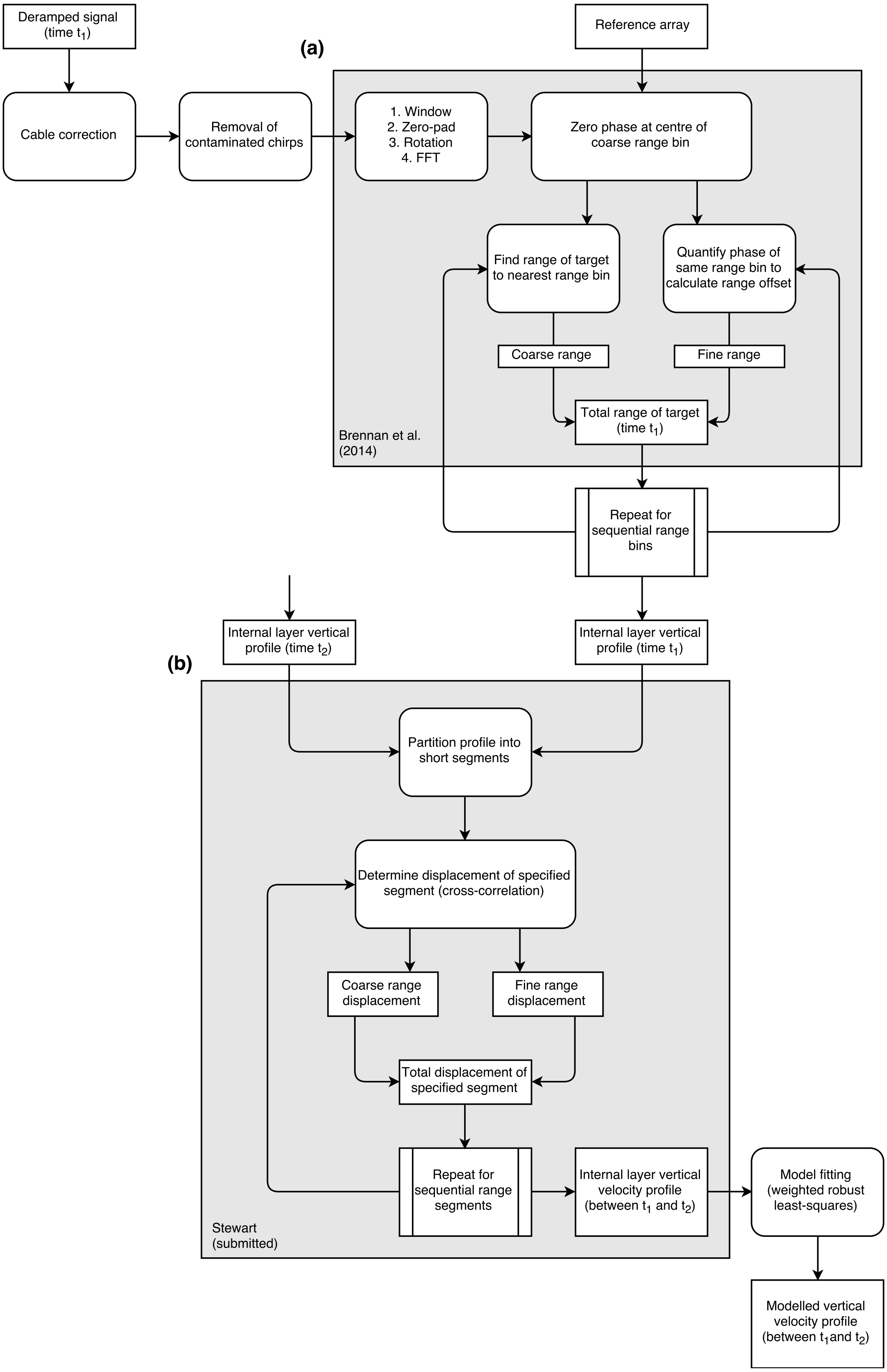

Figure S3. Schematic of ApRES processing steps to measure and model vertical velocities of internal layers. (a) Processing steps developed in *Brennan et al.* [2014]. (b) Processing steps developed in *Stewart* [2018]. The rest of the processing steps were developed in this study.

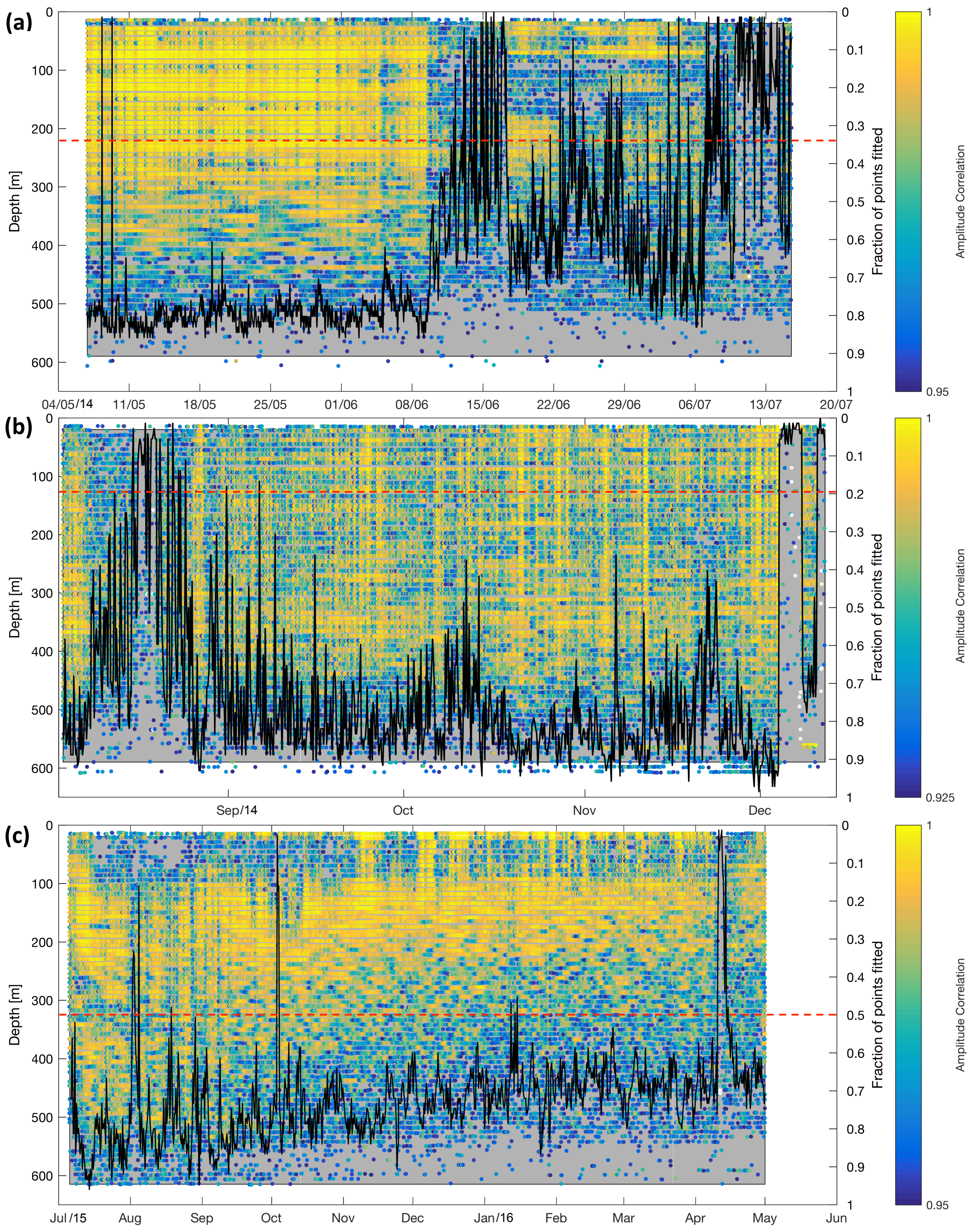

Figure S4. Coherence strength from cross-correlation of internal reflector pairs and the fraction of internal layers within each profile used in subsequent analyses, measured with ApRES at S30 at sites (a) 14a; (b) 14b; and (c) 15. All reflector pairs falling below the required correlation threshold (lower limit of the color bars) were excluded from further analysis. The depth range used for analyses is indicated in grey. The threshold for overall profile inclusion is indicated in dashed red, where, if the fraction of points fitted falls below this threshold, the entire profile is excluded altogether from further analysis. Note the different color scales used.

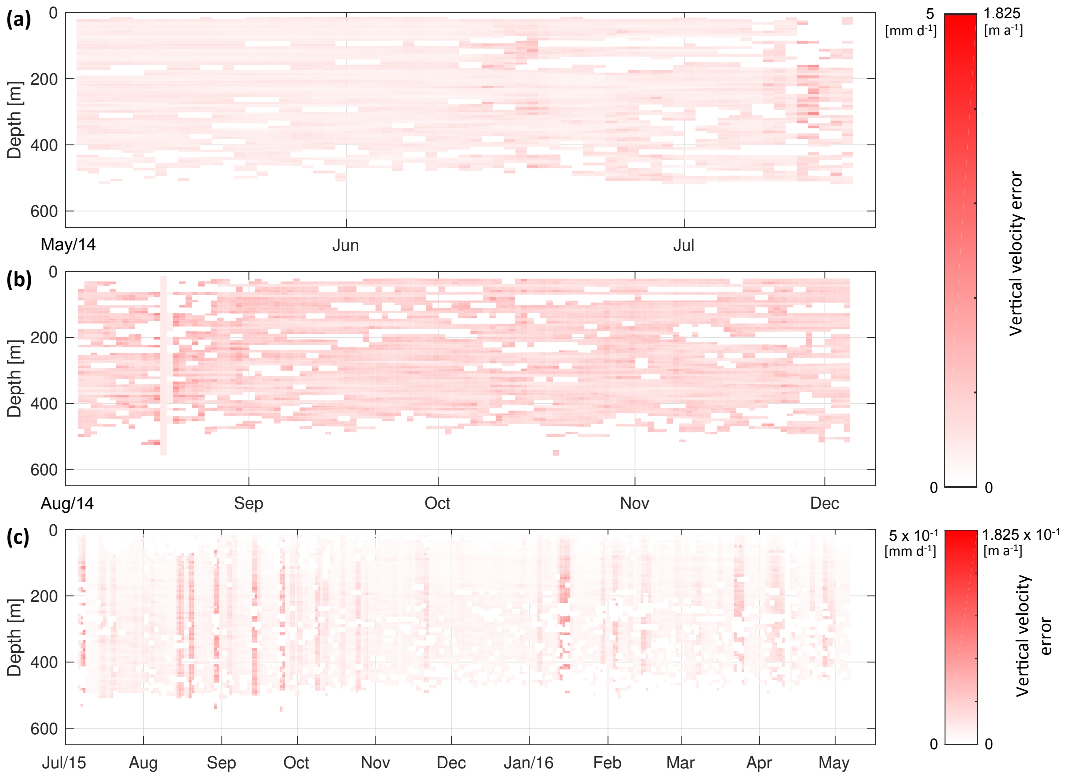

Figure S5. Standard error of internal reflector vertical velocity time series relative to the transmitting/receiving antenna within the entire ice column measured using ApRES at S30 from deployments (a) 14a; (b) 14b; and (c) 15. The effects of surface ablation on internal reflector vertical velocities were removed. Note the different scales used for time and value between subplots.

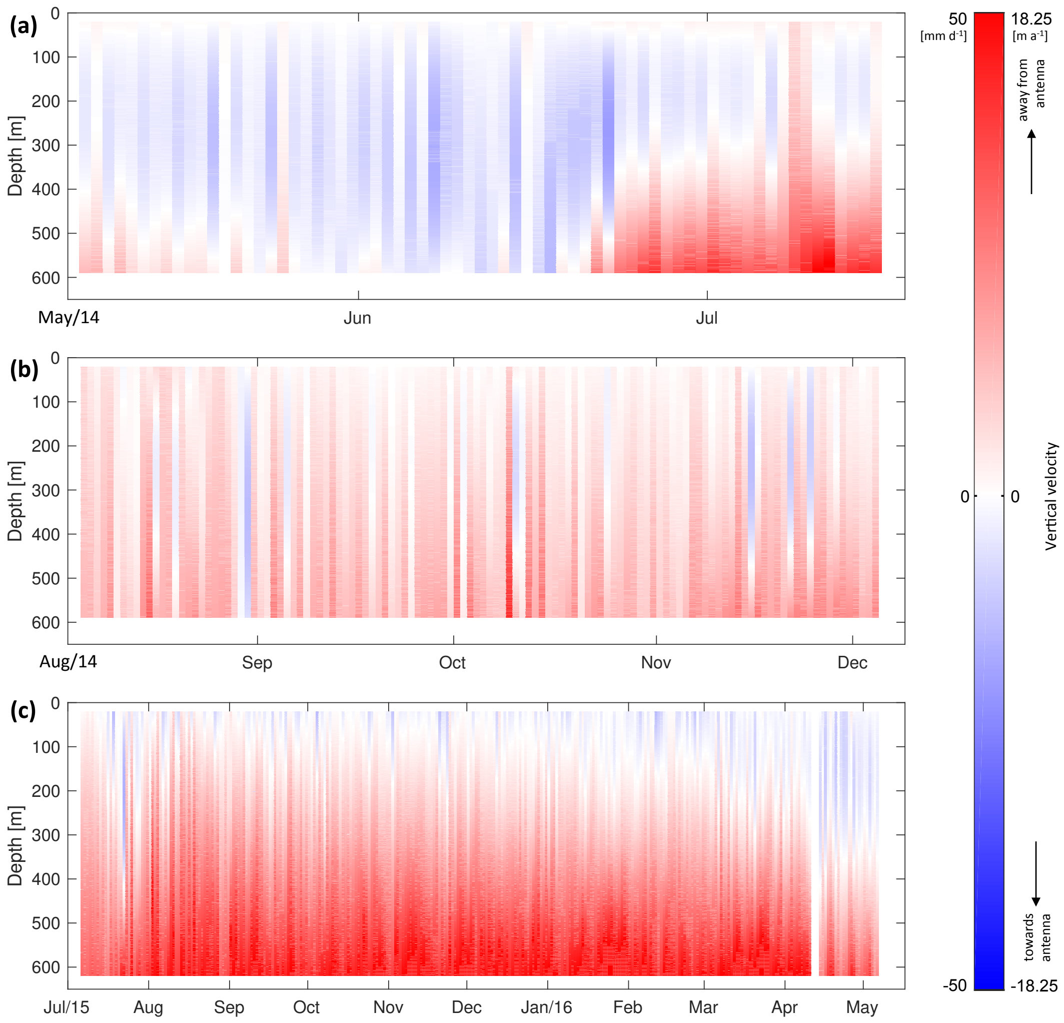

195 **Figure S6.** Time series of modeled internal layer vertical velocity profiles within the ice column measured using ApRES at S30 at sites (a) 14a; (b) 14b; and (c) 15. All profiles were automatically fitted with a quadratic model (Eq. 10). Here, blue represents upward movement of an internal reflector, and red downward movement. Note the different scales used for time and distance between subplots.
