# Supplementary figures and images for: Physical Conditions of Fast Glacier Flow: 3. Seasonally‐Evolving Ice Deformation on Store Glacier, West Greenland
Source: J Geophys Res Earth Surf. 2019 Jan 30;124(1):245–67. doi: 10.1029/2018JF004821 (PMC6472443; doi:10.1029/2018JF004821)

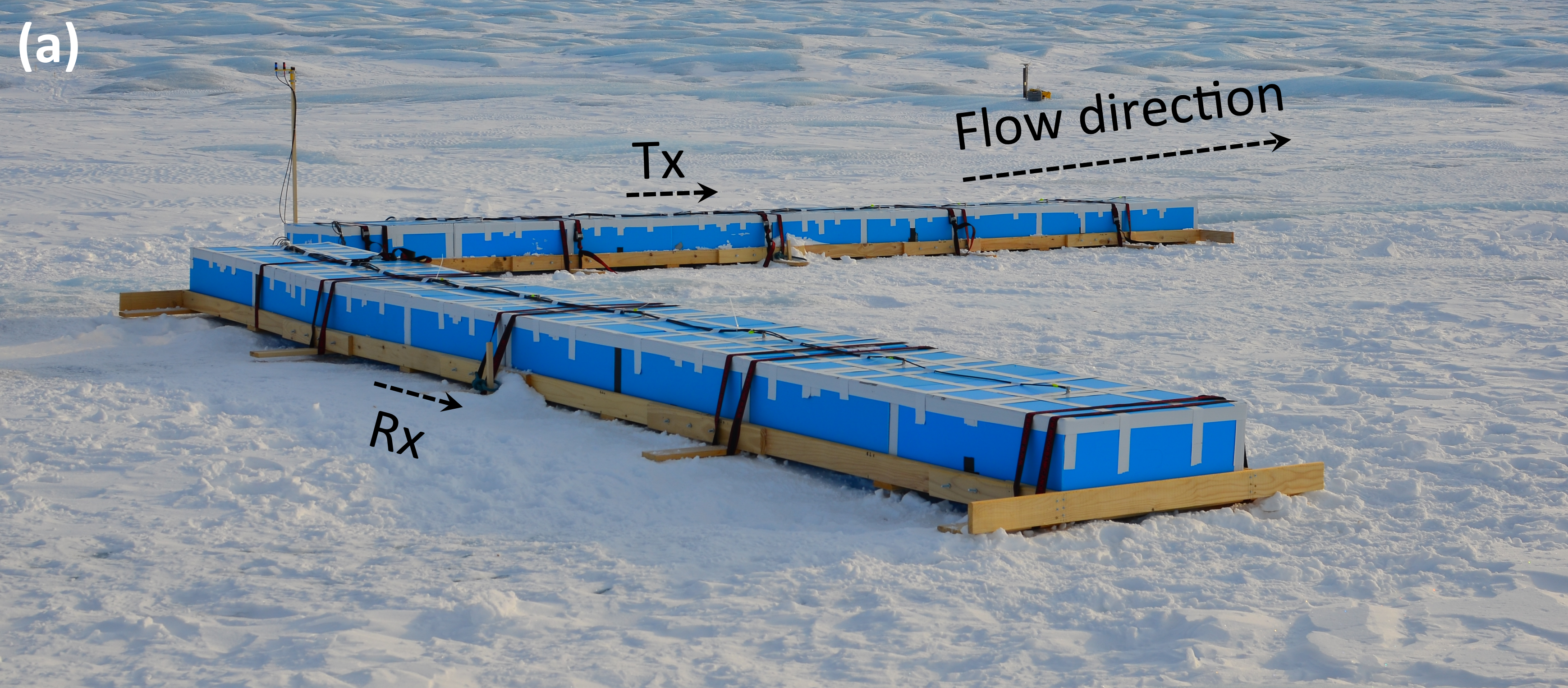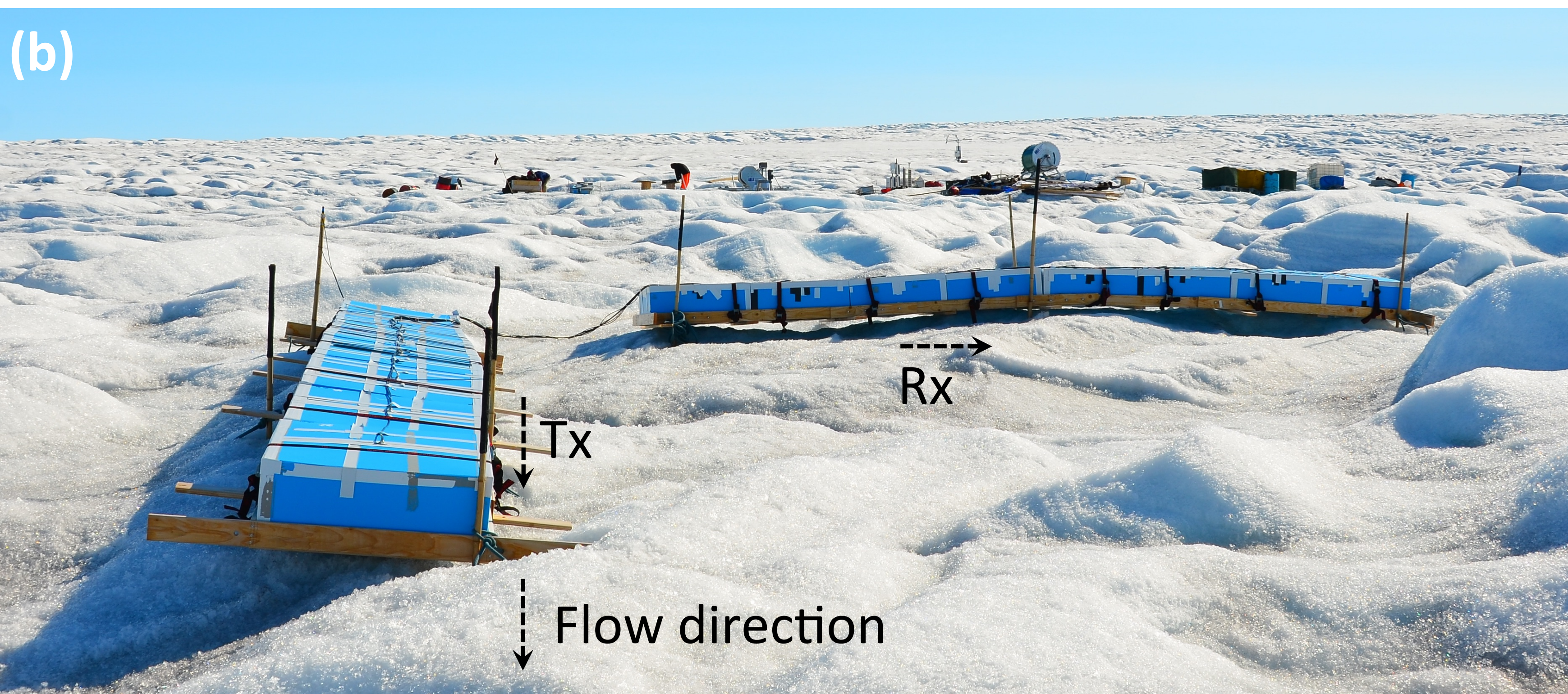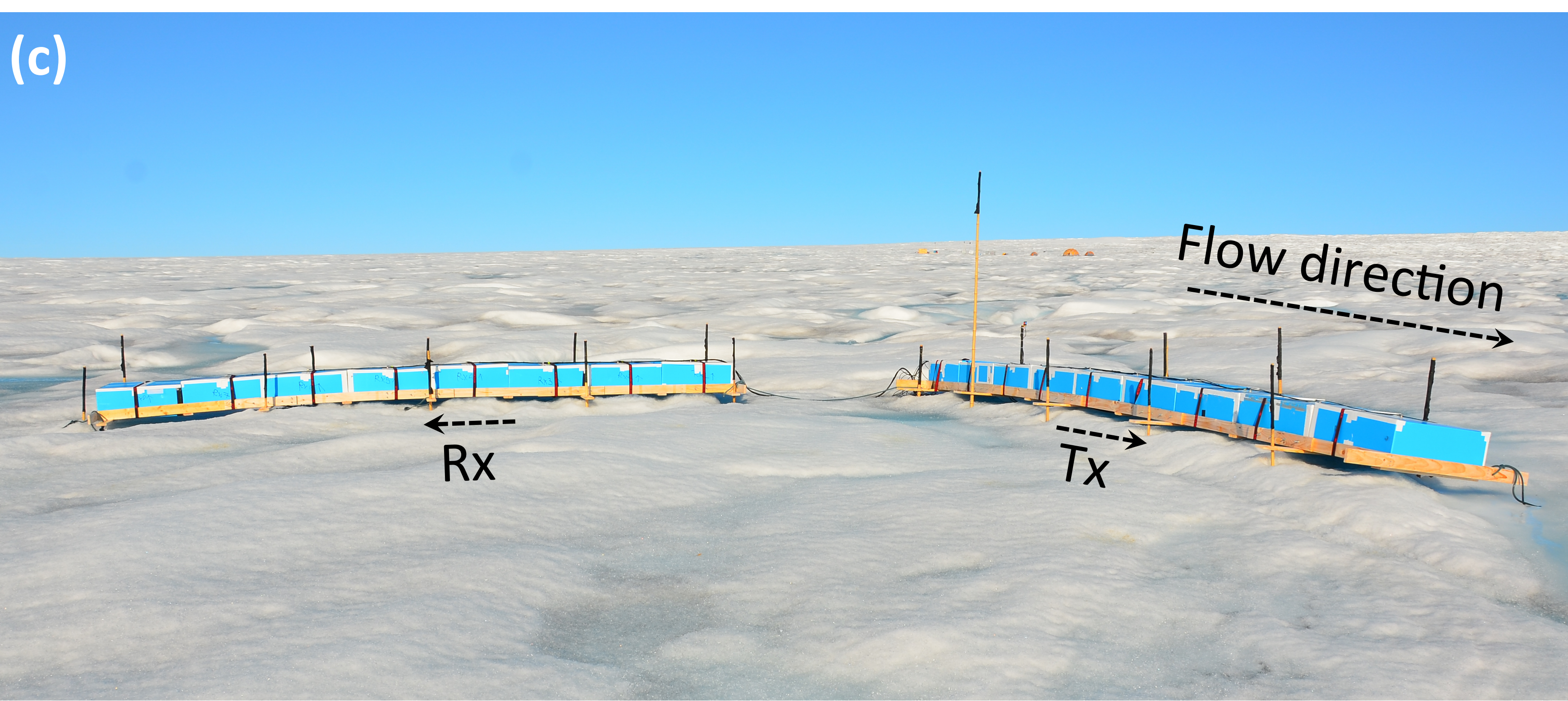

Supplement: Supplementary file 2 — Figure S1 [file JGRF-124-245-s002.pdf]

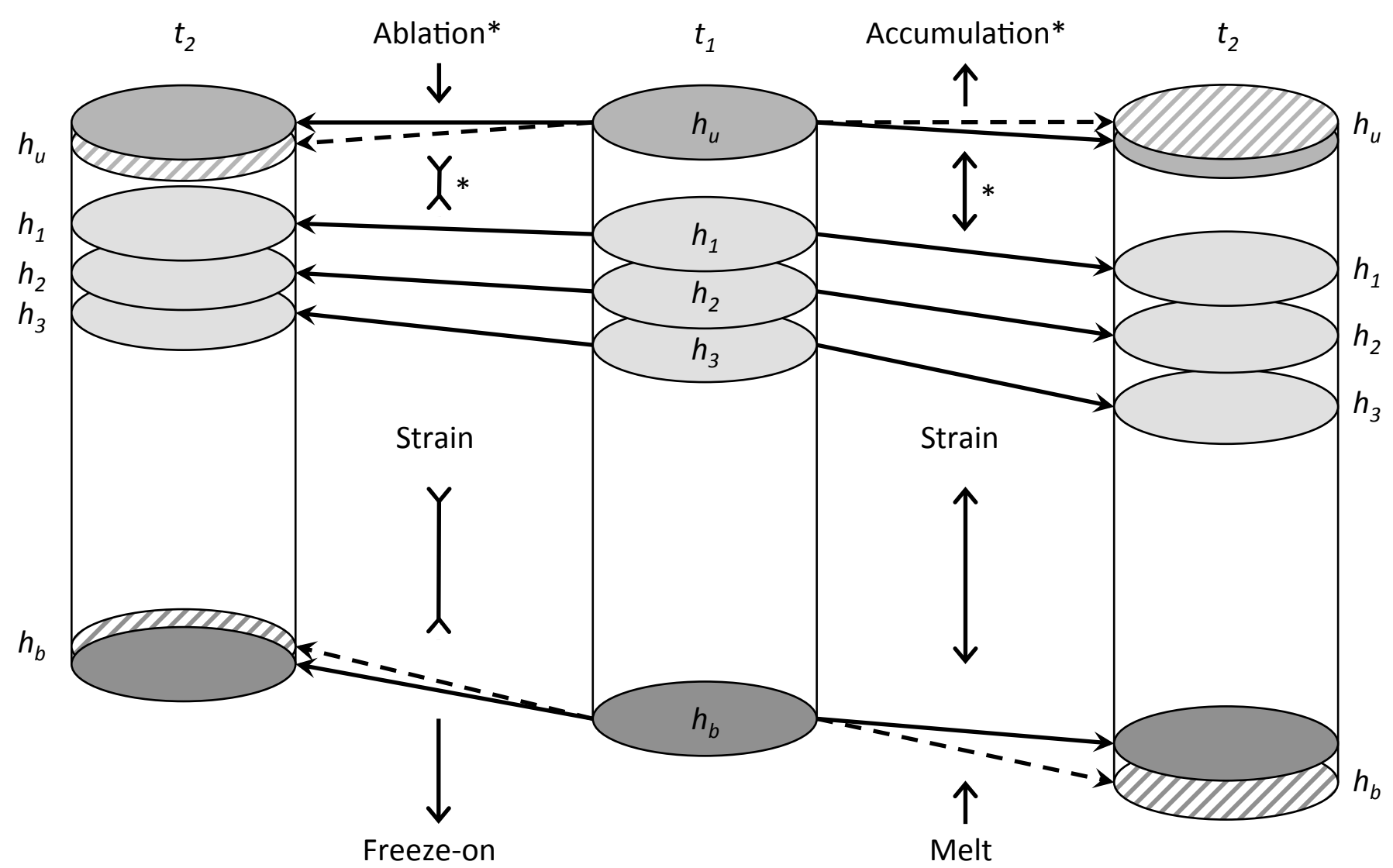

Supplement: Supplementary file 3 — Figure S2 [file JGRF-124-245-s003.pdf]

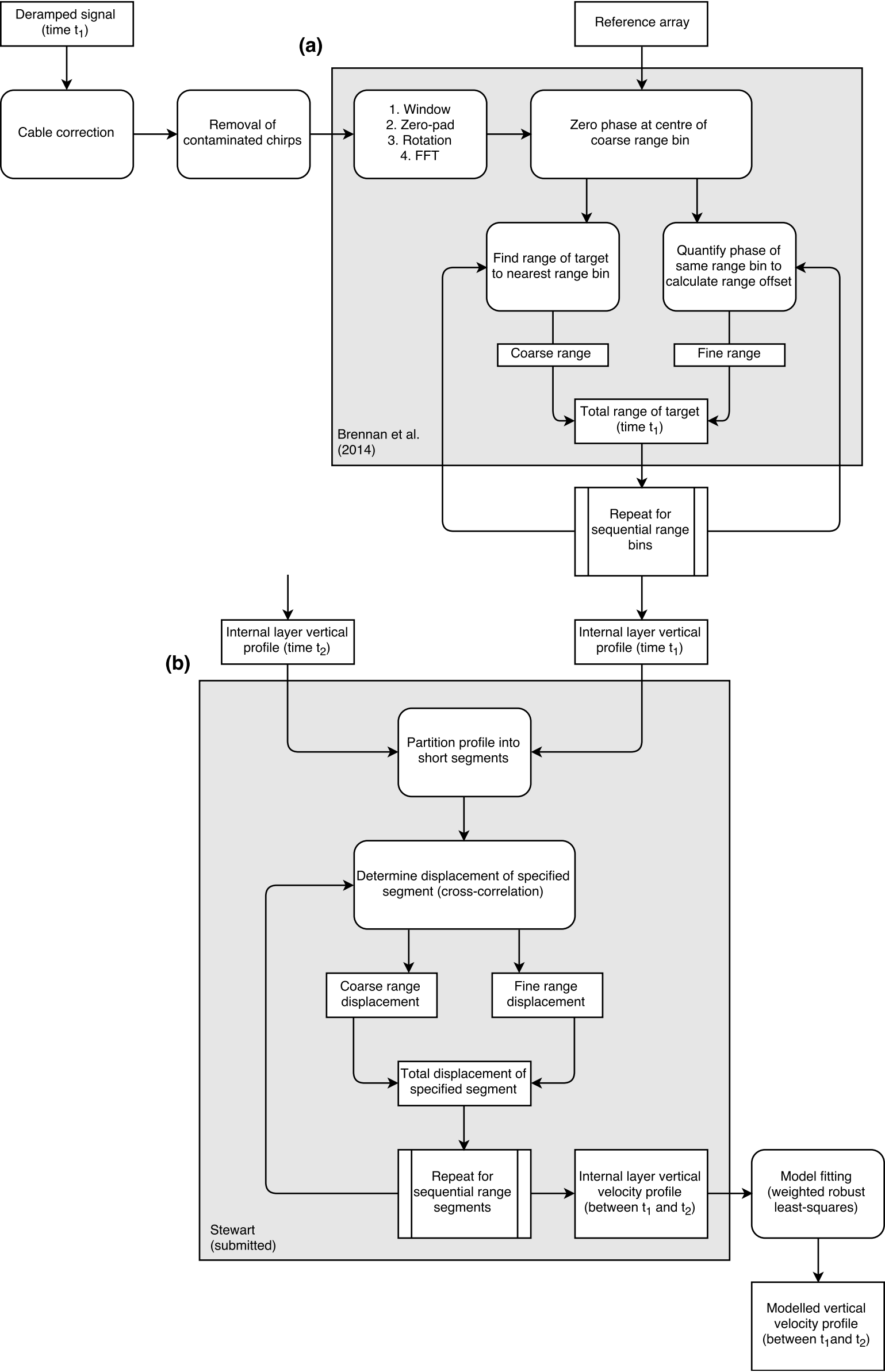

Supplement: Supplementary file 4 — Figure S3 [file JGRF-124-245-s004.pdf]

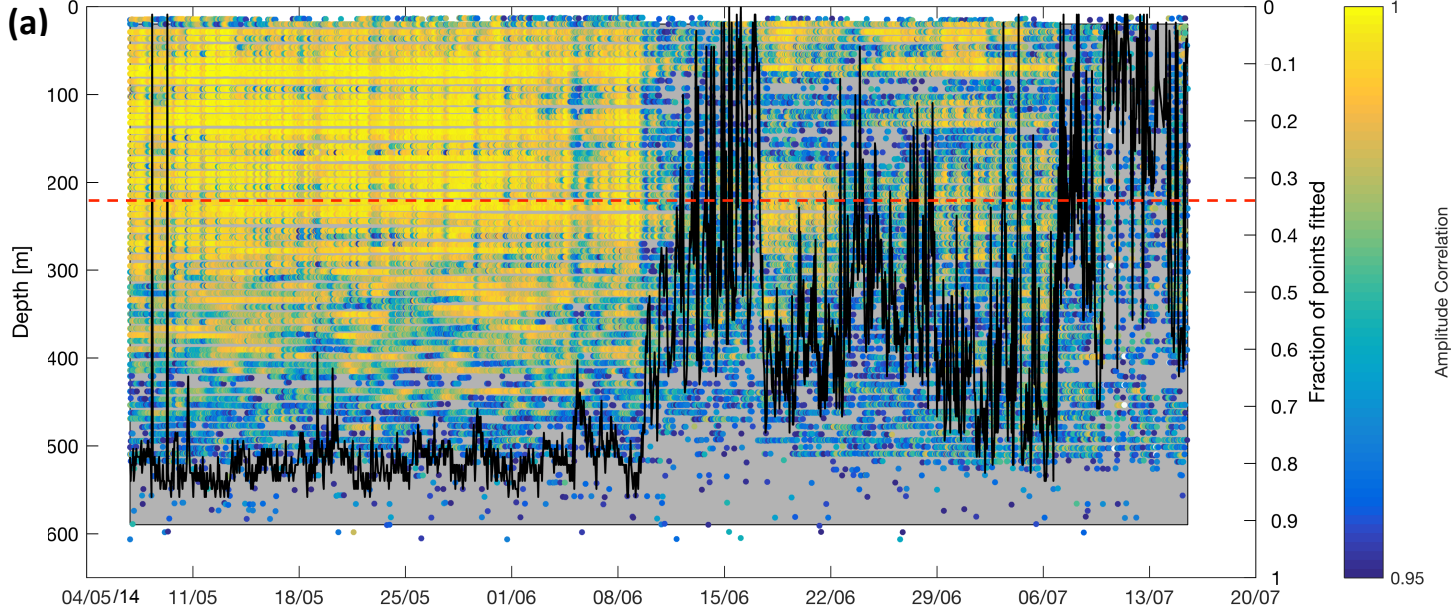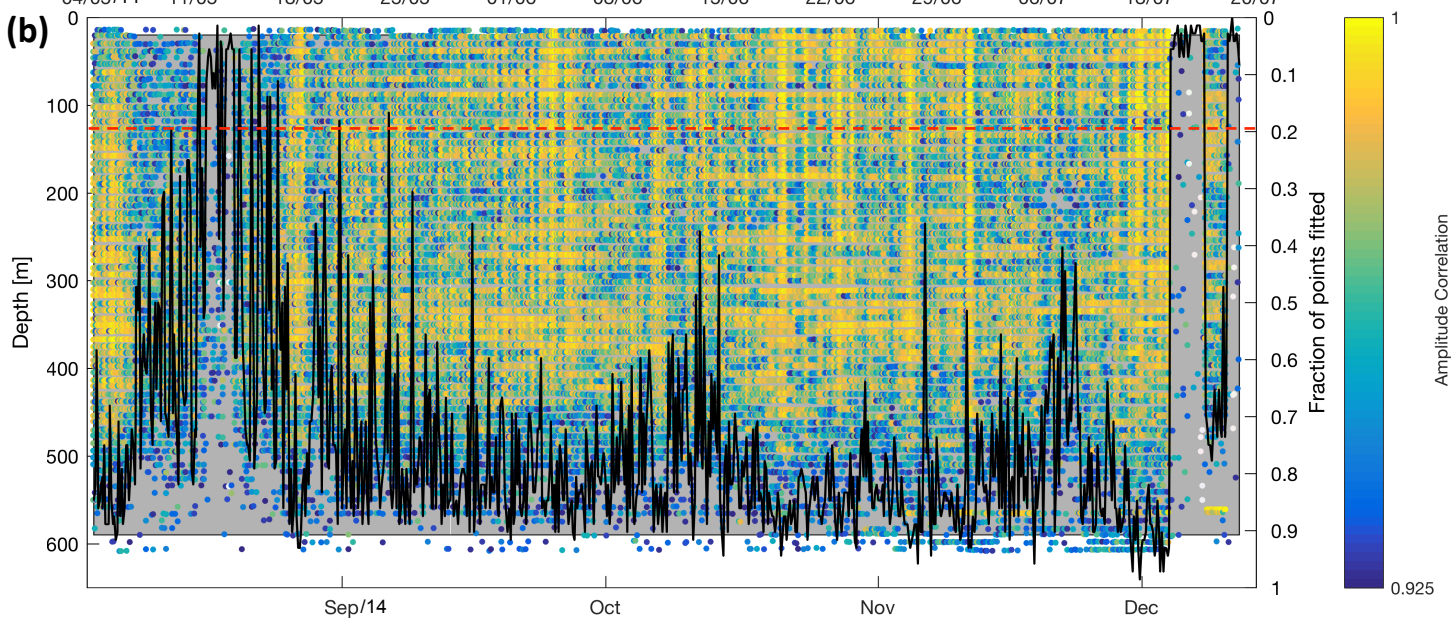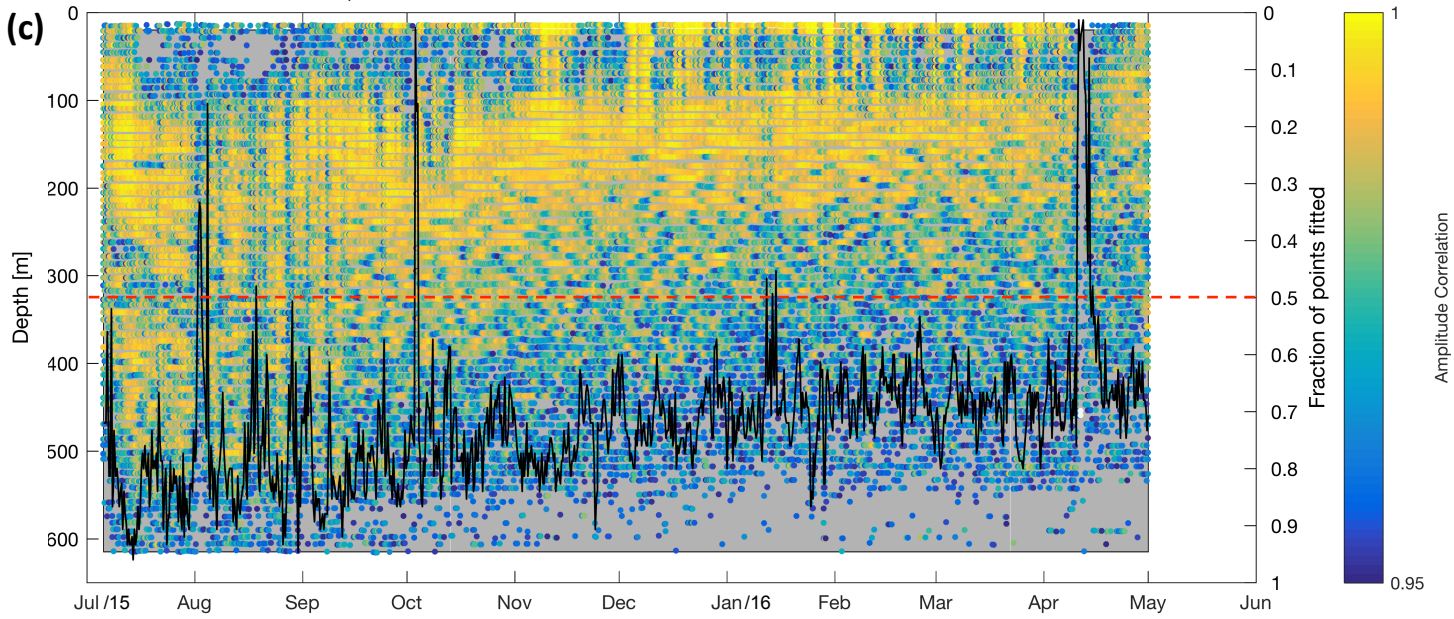

Supplement: Supplementary file 5 — Figure S4 [file JGRF-124-245-s005.pdf]

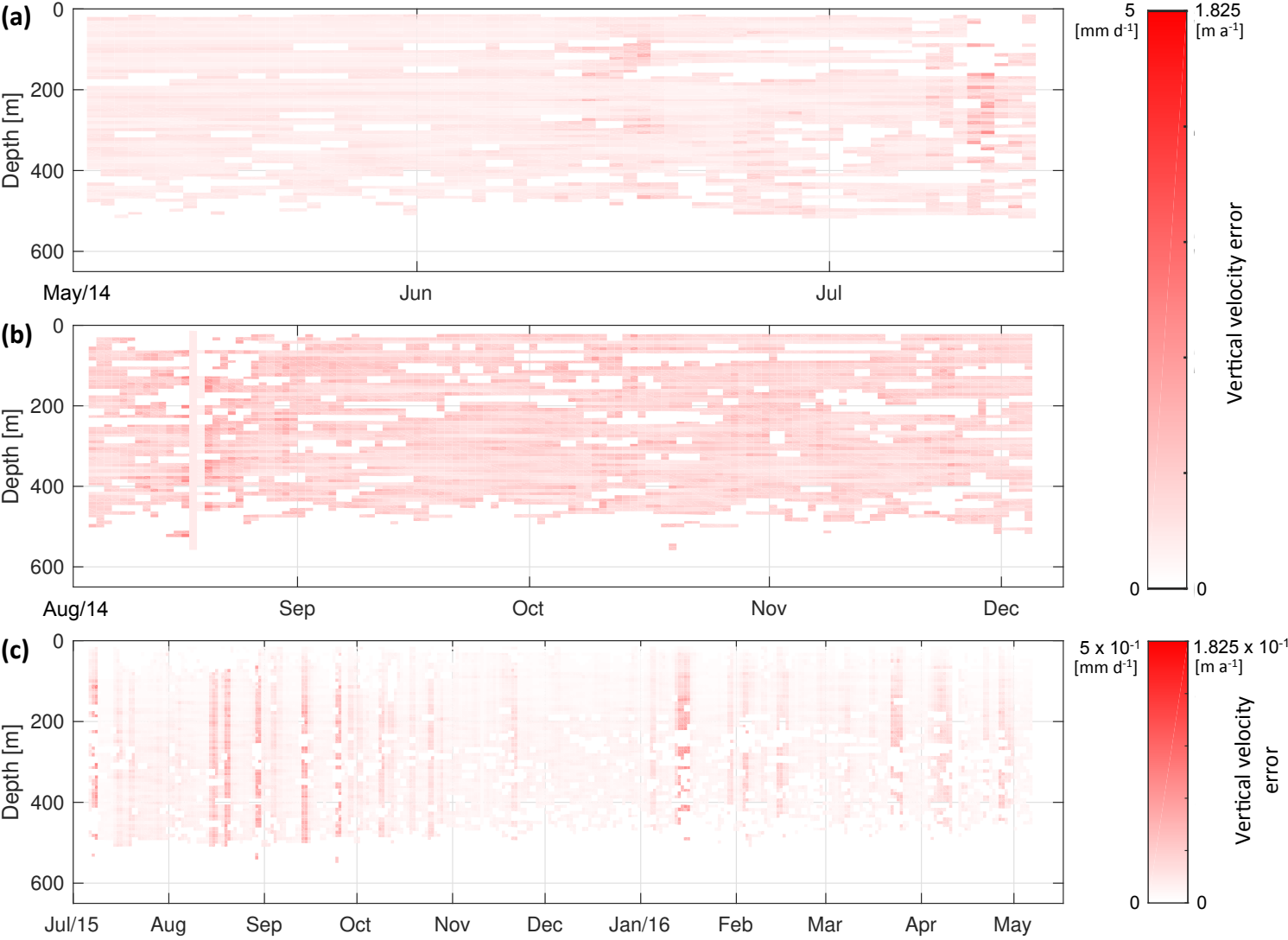

Supplement: Supplementary file 6 — Figure S5 [file JGRF-124-245-s006.pdf]

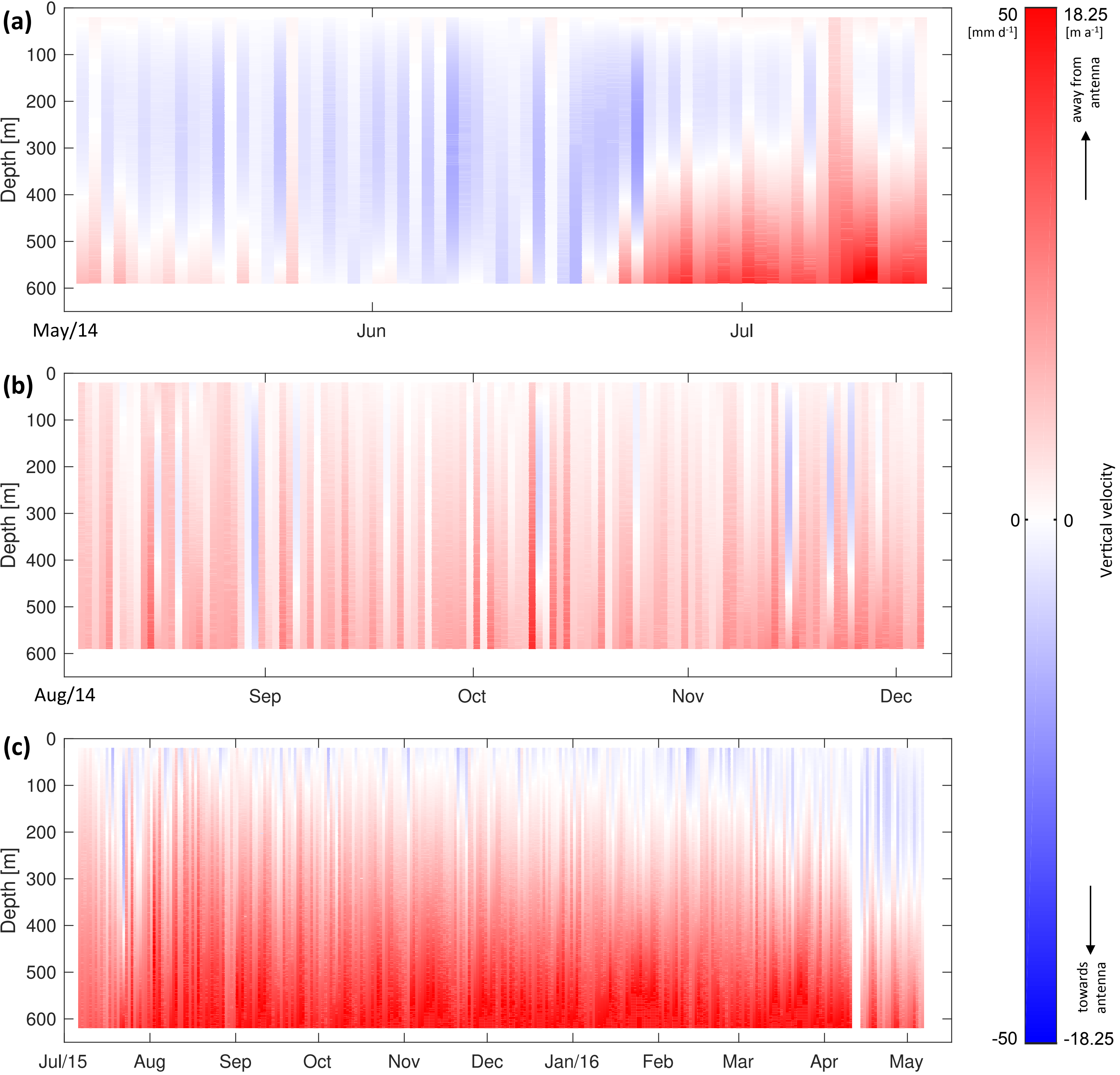

Supplement: Supplementary file 7 — Figure S6 [file JGRF-124-245-s007.pdf]
